# Host Cell Amplification of Nutritional Stress Contributes To Persistence in Chlamydia trachomatis

**DOI:** 10.1128/mbio.02719-22

**Published:** 2022-11-15

**Authors:** Nick D. Pokorzynski, Monisha R. Alla, Rey A. Carabeo

**Affiliations:** a Department of Pathology and Microbiology, University of Nebraska Medical Center, Omaha, Nebraska, USA; University of California, San Francisco; New York University School of Medicine

**Keywords:** host-pathogen interactions, intracellular pathogens, nutrient starvation, stress response, transcription

## Abstract

Persistence, a viable but non-replicating growth state, has been implicated in diseases caused by Chlamydia trachomatis. Starvation of distinct nutrients produces a superficially similar persistent state, implying convergence on a common intracellular environment. We employed host-pathogen dual RNA-sequencing under both iron- and tryptophan-starved conditions to systematically characterize the persistent chlamydial transcriptome and to define common contributions of the host cell transcriptional stress response in shaping the intracellular environment. The transcriptome of the infected host cells was highly specific to each nutritional stress, despite comparable effects on chlamydial growth and development in each condition. In contrast, the chlamydial transcriptomes between nutritional conditions were highly similar, suggesting some overlap in host cell responses to iron limitation and tryptophan starvation that contribute to a common persistent phenotype. We demonstrate that a commonality in the host cell responses is the suppression of GTP biosynthesis, a nucleotide for which Chlamydia are auxotrophic. Pharmacological inhibition of host IMP dehydrogenase (IMPDH1), which catalyzes the rate-limiting step in *de novo* guanine nucleotide synthesis, resulted in comparable GTP depletion to both iron and tryptophan starvation and induced chlamydial persistence. Moreover, IMPDH1 inhibition and iron starvation acted synergistically to control chlamydial growth. Thus, host cell reduction in GTP levels amplifies the nutritional stress to intracellular chlamydiae in infection-relevant models of persistence, illustrating the determinative role the infected host cell plays in bacterial stress responses.

## INTRODUCTION

The dynamics of intracellular infection reflect the interaction of the pathogen and the host cell, with the outcome of the battle shaped by the competition between pathogen virulence and host counteractive measures (1). A cytokine that tilts the balance toward the host is interferon-gamma (IFNγ), the effects of which are amplified by the JAK/STAT signaling pathway to induce a varied collection of responsive genes, including several anti-microbial effectors (2). In turn, pathogens have evolved to acquire strategies that attenuate or neutralize IFNγ (3). A primary mechanism by which IFNγ inhibits pathogen replication is the withholding of critical nutrients, such as molecular iron, contributing to a process known as nutritional immunity (4). Importantly, iron is also an important nutrient for the host cell, and its depletion to combat intracellular infection likely results in the induction of additional pathways which may or may not impact the pathogen (5, 6). A similar scenario applies to tryptophan depletion mediated by the IFNγ-inducible catabolizing enzyme indoleamine-2,3-dioxygenase (IDO1) (7). While it starves intracellular pathogens for tryptophan, it concomitantly deprives the host of this essential amino acid. Here, we interrogate the response of the infected host cell subjected to models of nutrient starvation (e.g., iron starvation by chelation with 2,2-bipyridyl and tryptophan limitation via growth in tryptophan-depleted medium) typically used to simulate specific IFNγ-responsive anti-microbial effectors. Specifically, we sought to determine whether the effects of nutrient starvation on an intracellular bacterial pathogen are conditioned by the response of the host cell to the same primary insult.

A typical response of the Gram-negative, obligately intracellular bacterial pathogen Chlamydia trachomatis (*Ctr*), to iron or tryptophan starvation is the establishment of “persistence”. *Chlamydiae* are distinguished by a biphasic developmental cycle that interconverts an infectious, non-replicative elementary body (EB) with a noninfectious, replicative reticulate body (RB) (8). *Chlamydiae* can disengage their normal developmental program and enter a persistent state in response to a wide array of stress (9, 10), including antibiotic treatment (11), amino acid starvation (12–14), or biometal limitation (15–17). Chlamydial persistence has been suggested to be clinically relevant as persistent *Chlamydiae* are re-activatable (18) and tolerant to bactericidal antibiotics (19, 20). Thus, dormant, persistent chlamydiae may resist standard antibiotic regimens, allowing acute symptoms to reemerge after the pathogen resumes its normal developmental cycle (10).

**FIG 1:**
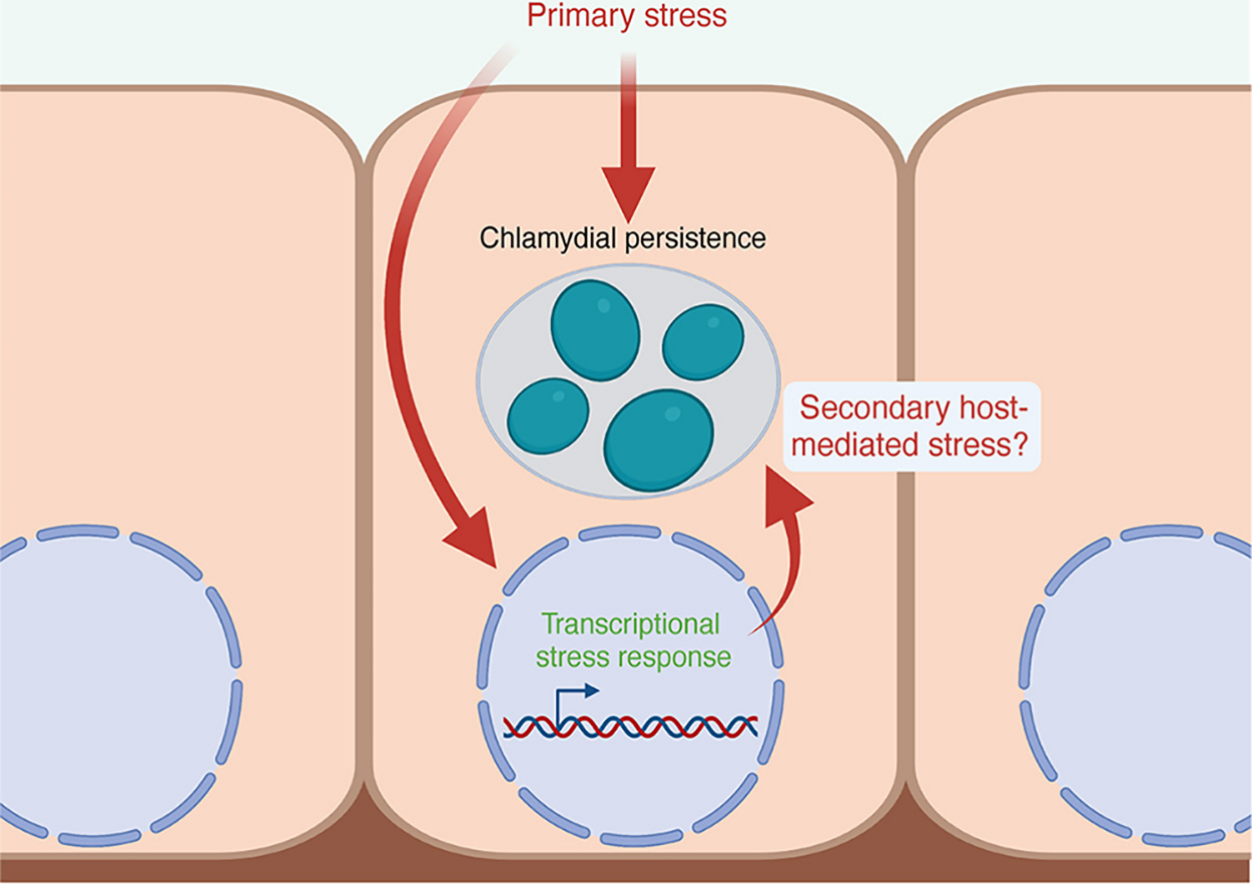
Model of the contribution of secondary host-mediated stresses that may impact chlamydial persistence. The impact of a primary stress (nutritional, immunological, or otherwise) on a Chlamydia-infected eukaryotic cell can be understood both by how it effects the intracellular bacteria and how it effects the host cell. However, the response of the host cell to the primary stress may trigger subsequent effects on the intracellular bacteria. These secondary, host-mediated stresses may have important roles in the character of bacterial stress responses when residing in a host cell. Created with BioRender.com.

The prevailing view is that persistence is the result of the accumulated effects of the stressor on the pathogen. For example, persistence resulting from iron starvation is thought to arise from the combined action of inactivating iron-dependent enzymes and the dysregulation of the iron-responsive regulon, both of which are expected to have pleiotropic effects on Chlamydia (15, 21). Tryptophan starvation on the other hand is expected to reduce translation of proteins that are tryptophan-rich; and the resulting skewed proteome disrupts chlamydial growth and development (22). These are likely to be an oversimplification of the interaction between pathogen and host because the effects of the nutritional stress on the host cell, which itself deploys adaptive responses, are not considered (Fig. 1). In other words, the nature of these adaptive responses might inform on the host cell priority, i.e., inhibit pathogen growth or survive the side effects of the anti-microbial effectors. It is also possible that these 2 priorities can coexist, and perhaps cooperate to clear infection effectively.

We therefore systematically compared tryptophan- and iron-starved *Ctr*-infected epithelial cells via host-pathogen dual RNA-sequencing (RNA-seq). We find that the transcriptome of the infected host cell is distinct to the nutritional stress applied, despite a high degree of similarity in the morphological, developmental, and transcriptional outcomes for the resident chlamydiae. In addition to resolving a “core” persistent chlamydial transcriptome that is induced irrespective of the stress condition, we also find a progressive, accessory subset of the persistent transcriptome that is unique to each stress condition, implying an active response by the pathogen. By dissecting the relationship between the host and pathogen transcriptional response, we unexpectedly discover that persistent *Ctr* respond to host-mediated depletion of GTP, a nucleotide for which Chlamydia are auxotrophic. This was a common metabolic consequence of both iron- and tryptophan starvation of host cells, regardless of infection. The pathogen responded accordingly by the enhanced transcription of genes for biosynthesizing the GTP-dependent cofactors, riboflavin, and tetrahydrofolate (THF). Treatment of infected cells with mizoribine, a specific inhibitor of IMPDH1, reproduced both the growth defect and the chlamydial upregulation of riboflavin and THF biosynthesis genes. Together, these data support the view that a nutritionally stressed host cell produces secondary, compounding, and anti-bacterial effects on invading pathogens. That GTP depletion is a common metabolic consequence of the 2 stresses indicate that amplification of the primary stress may explain the highly similar response of Chlamydia to distinct nutritional stress. We argue that the ability of the host cell to amplify primary stress should be considered as an important component of the broader phenomenon of nutritional immunity.

## RESULTS

### The transcriptional response of Chlamydia-infected epithelial cells is dependent on the nutritional condition.

**FIG 2:**
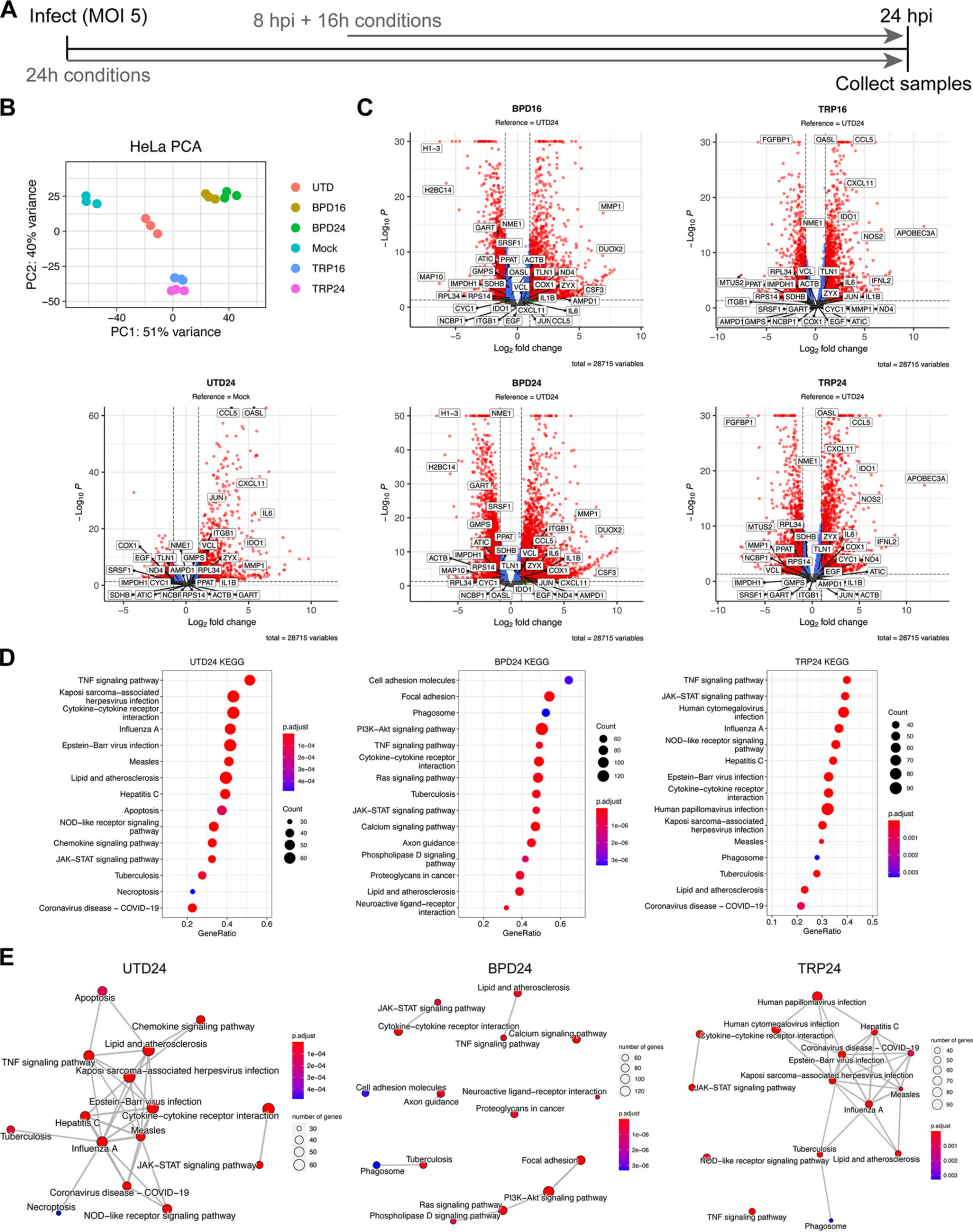
The infected host cell transcriptome differs between models of chlamydial persistence. (A) Diagram depicting the experimental design used throughout this study, where 24h treatment conditions (UTD24, BPD24, TRP24) begin at the time of infection, and 16h treatments begin at 8 h postinfection (BPD16, TRP16). (B) Principal-component analysis (PCA) of the HeLa transcriptomes derived from the various experimental conditions. All data are N = 3. (C) Volcano plots of the differentially expressed (DE) genes under each persistence-inducing condition (adjusted *P*-value < 0.05, |Fold change| > 2.0). Note that UTD24 is relative to the mock-infected condition (Mock) whereas all other conditions are relative to UTD24. Red, DE, blue, *P < *0.05, |FC| < 2.0, green, *P > *0.05, |FC| > 2.0, gray, *P > *0.05, |FC| < 2.0. (D) Dot plots for the 15 most enriched pathways identified by clusterProfiler in UTD24, BPD24 and TRP24. Dot size reflects the number of genes enriched in the pathway and dot color indicates the statistical significance of pathway enrichment. (E) Enrichment network map for the 15 most enriched pathways identified by clusterProfiler in UTD24, BPD24 and TRP24. Dot size and color are the same as in (D).

To establish models of chlamydial persistence, we subjected *Ctr*-infected HeLa cells to iron starvation, by treatment with the membrane permeable iron chelator 2,2-bipyridyl (BPD) (16), or tryptophan starvation, by culturing in a defined medium lacking tryptophan (TRP) (23, 24). We applied 2 treatment regimens (Fig. 2A and Table 1), one which started at the time of infection and continued for 24 h (h; BPD24, TRP24), reflecting established models of chlamydial persistence, or one that began at 8h postinfection (hpi) and continued for 16 h (BPD16, TRP16), thereby allowing *Ctr* to establish a productive infection and differentiate into the replicative RB state prior to nutrient starvation. We hypothesized that the transcriptional response of *Ctr*-infected epithelial cells to nutritional stress could reveal underlying mechanisms that contribute to the establishment of chlamydial persistence. We therefore implemented dual RNA-seq to resolve the host and pathogen transcriptomes during iron or tryptophan starvation. Our experiment was designed to capture the presumably small fraction of chlamydial transcripts produced in persistently infected HeLa cells, and we accordingly used a multiplicity of infection (MOI) of 5 for each condition. In agreement with recent reports, we recovered high levels of chlamydial transcripts in each library (25), with no fewer than 7.5 × 10^6^ mapped reads under any condition. We also note that across replicates, no more than 0.01% of the total library mapped to the chlamydial reference genome in any mock-infected sample, indicating a negligible influence of cross-aligned reads in our samples. Dual RNA-seq produced HeLa transcriptomes with no fewer than 3.3x10^7^ mapped reads in any condition.

**TABLE 1:** Description of host-pathogen dual RNA-sequencing conditions and statistical comparisons

HeLa RNA-seq			C. trachomatis RNA-seq		
Conditions	Identifier	Comparisons	Conditions	Identifier	Comparisons
Mock-infected	Mock	Reference			
Untreated, infected 24 hpi	UTD24	UTD24:Mock, Reference	Untreated, infected 24 hpi	UTD24	Reference
Infected 8 hpi + 16 h bipyridyl treatment	BPD16	BPD16:UTD24	Infected 8 hpi + 16 h bipyridyl treatment	BPD16	BPD16:UTD24
Infected 8 hpi + 16 h tryptophan starvation	TRP16	TRP16:UTD24	Infected 8 hpi + 16 h tryptophan starvation	TRP16	TRP16:UTD24
Infected 0 hpi + 24 h bipyridyl treatment	BPD24	BPD24:UTD24	Infected 0 hpi + 24 h bipyridyl treatment	BPD24	BPD24:UTD24
Infected 0 hpi + 24 h tryptophan starvation	TRP24	TRP24:UTD24	Infected 0 hpi + 24 h tryptophan starvation	TRP24	TRP24:UTD24

Principal-component analysis of the HeLa transcriptomes revealed that sample clustering was dependent on the treatment condition applied, with BPD and TRP forming independent clusters distinct from the untreated, infected group (UTD24) (Fig. 2B). All infected conditions clustered distinctly compared to the mock-infected control group (Mock). Subsequent analysis of differential gene expression (adjusted *P*-value < 0.05, |FC| > 2) was performed by analyzing UTD24 in reference to Mock, whereas all nutrient-starved samples were analyzed in reference to UTD24 to account for the influence of infection (Table 1). Complete details of the differential expression analysis can be found in Data set S1. We observed that compared to UTD24, all treatment conditions altered the global transcriptional profile, resulting in a more significantly downregulated portion of the DE genes (Fig. 2C). Whereas UTD24 resulted in a downregulated set of 406 genes compared to Mock, no fewer than 1297 genes were significantly downregulated under any nutrient-deprived condition when compared to UTD24 (Data set S1). We note the significant upregulation of genes previously identified to respond to acute and persistent chlamydial infection, such as the antiviral protein *OASL*, in UTD24, TRP16, and TRP24 (Fig. 2C) (26). Having accounted for differentially expressed genes due to infection, the remaining differences in gene expression could be assigned confidently to the host response to nutritional stress or unique activities of persistent chlamydiae.

10.1128/mbio.02719-22.6 DATA SET S1 Results of DESeq2 differential gene expression analysis. Download Data Set S1, XLSX file, 14.6 MB. Copyright © 2022 Pokorzynski et al. 2022 Pokorzynski et al. https://creativecommons.org/licenses/by/4.0/ This content is distributed under the terms of the Creative Commons Attribution 4.0 International license.

Next, we performed KEGG pathway gene set enrichment analysis (GSEA) to identify differentially regulated pathways between conditions. Due to the similarity between treatment regimens (Fig. 2B), here we display only the results from the analysis of the 24 h conditions but results for all conditions are provided in Data set S2. We sorted pathways based on their enrichment score and plotted the results for the 15 most enriched pathways (Fig. 2D and E). We found that both BPD24 and TRP24 shared with UTD24 the enrichment of pathways related to infection, but also displayed uniquely enriched pathways – most notably in BPD24. The most enriched pathways in BPD24 comprised various categories, such as “Focal adhesion” (hsa04510), “PI3K-Akt signaling pathway” (hsa04151), and “Calcium signaling pathway” (hsa04020) (Fig. 2D). We next inferred relatedness of the various enriched pathways by generating enrichment network maps for each condition (Fig. 2E). We found that both UTD24 and TRP24 produced highly interconnected networks that form nodes related to the response to infection. In contrast, BPD24 produced a disconnected network, indicating functionally disjointed iron-responsive biological processes were induced under this condition. These data suggest that intracellular bacteria such as *Ctr* will encounter highly disparate host cell responses during nutritional stress, which would be expected to have varying influences on the growth and development of resident chlamydiae.

10.1128/mbio.02719-22.7 DATA SET S2 Results of clusterProfiler gene set enrichment KEGG pathway analysis. Download Data Set S2, XLSX file, 0.07 MB. Copyright © 2022 Pokorzynski et al. 2022 Pokorzynski et al. https://creativecommons.org/licenses/by/4.0/ This content is distributed under the terms of the Creative Commons Attribution 4.0 International license.

### Distinct nutritional stressors produce phenotypically similar persistent states in Chlamydia trachomatis.

**FIG 3:**
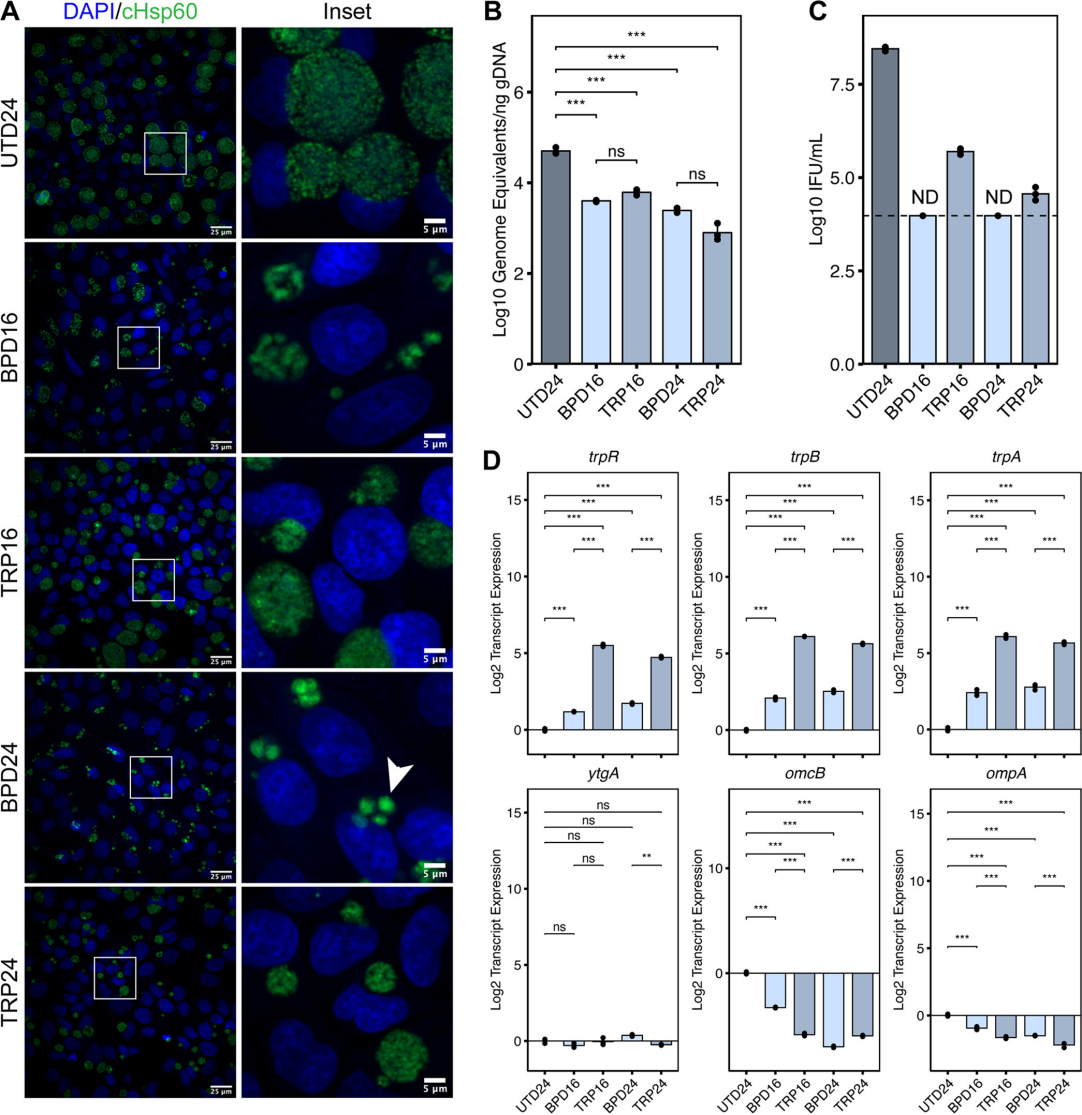
Alternative models of chlamydial persistence exhibit common phenotypes. (A) Immunofluorescent confocal microscopic analysis of chlamydial morphology under various models of chlamydial persistence. Micrographs are representative of at least 3 independent biological replicates (N = 3). Chlamydial organisms were detected by immunostaining against the cytosolic Hsp60 homologs, GroEL_1-GroEL_3. Nuclei were detected by staining with DAPI. Arrowheads indicate aberrantly enlarged bacteria. (B) Determination of genome equivalents by quantitative PCR against the *euo* locus under the various persistence models. (C) Measurement of infectious progeny generation during nutritional stress in a reinfection assay. Dotted line indicates calculated limit of detection for the assay. ND, not detected. (D) Gene expression profiles for various nutritionally- or developmentally-regulated chlamydial genes. All plots represent the mean and standard deviation of three independent biological replicates (N = 3). Statistical significance in all panels was determined by one-way ANOVA followed by Tukey’s *post hoc* test of honestly significant differences (two-tailed). *, *P < *0.05, **, *P < *0.01, ***, *P < *0.001, ns, not significant.

Despite marked differences in the infected host cell transcriptional response to iron or tryptophan starvation, chlamydial persistence is not typically differentiated between various experimental models. Thus, we assayed several physiological hallmarks of chlamydial persistence to discern the degree of similarity between different nutritional insults and treatment regimens. We first assayed chlamydial morphology by immunofluorescent confocal microscopy (Fig. 3A). In comparison to the untreated control (UTD24), all treatments produced qualitatively smaller inclusions, implying inhibited growth. We observed clear morphological differences between BPD and TRP, with BPD inclusions being occupied by aberrantly enlarged organisms while TRP inclusions displayed an “indiscrete” morphology, obscuring the observation of individual bacteria. We then analyzed genome copy number under each condition and observed that while all treatments significantly reduced genome equivalents compared to UTD24, no differences were statistically distinguishable between BPD and TRP (Fig. 3B). In contrast, we found that TRP was more permissive to the generation of infectious progeny (Fig. 3C), as BPD reduced recoverable inclusion forming units (IFUs) below the calculated limit of detection. Thus, while a comparable number of genome equivalents, and by extension chlamydial organisms, exist under each treatment condition, whether those chlamydiae can complete their developmental cycle is influenced by the model of persistence employed. However, the directionality of each effect was the same between conditions, underscoring the universality of the persistent phenotype.

We then analyzed the expression of a panel of chlamydial genes commonly used to indicate developmental dysregulation by reverse transcription quantitative PCR (RT-qPCR): *trpRBA*, *ytgA*, *omcB*, and *ompA* (Fig. 3D). Consistent with previous reports, we observed that transcription of the tryptophan salvage operon, *trpRBA*, was significantly upregulated in both BPD and TRP, though TRP increased *trpRBA* expression significantly compared to BPD. This is due to dual regulation by the tryptophan-dependent transcriptional repressor TrpR and the iron- and tryptophan-dependent repressor YtgR (23, 24). In contrast, transcription of *ytgA*, encoding a periplasmic iron-binding protein, another gene regulated by YtgR (27), was not significantly altered by any treatment condition, despite prior reports of its iron- and tryptophan-dependent induction (16, 24). Here, we utilized a transcriptome-based normalization method (see Materials and Methods and Supplementary Note) (Fig. S1), rather than normalizing to genome equivalents. This indicates that while YtgR regulation of *trpRBA* serves to increase the expression of the operon relative to the total transcriptome, YtgR regulation of *ytgA* maintains a constant proportion of transcripts across various developmental or nutritional conditions. We confirm that the expression of *omcB*, a cysteine-rich outer membrane protein associated with differentiation to the EB stage and common biomarker of persistence, is downregulated by nutritional stress, as is the expression of *ompA*, the major outer membrane protein in *Ctr*. While some statistically distinguishable differences exist between stress conditions, we find that these are differences in magnitude but not the directionality of expression. Because we cannot normalize for the relative severity of iron chelation compared to media-defined tryptophan starvation, we conclude that both stimuli produce highly similar phenotypic profiles, though iron starvation may have unique consequences on chlamydial cell morphology and developmental progression. Importantly, there was little discernible difference in any phenotype assayed with respect to the treatment regimen, suggesting that the induction of persistent development in *Ctr* is insensitive to the time at which nutritional stress is applied.

10.1128/mbio.02719-22.1 FIG S1 Analysis of stable control genes by geNorm. (A) Table displaying the step-wise exclusion of genes with the lowest stability (highest M value) following geNorm analysis. (B) Plot of average gene stability (M) during the stepwise exclusion of unstable genes. (C) Plot of pairwise variation of remaining control genes during the stepwise exclusion of unstable genes. Download FIG S1, PDF file, 0.3 MB. Copyright © 2022 Pokorzynski et al. 2022 Pokorzynski et al. https://creativecommons.org/licenses/by/4.0/ This content is distributed under the terms of the Creative Commons Attribution 4.0 International license.

### Chlamydia initiates a common transcriptional program in response to distinct nutritional stressors.

**FIG 4:**
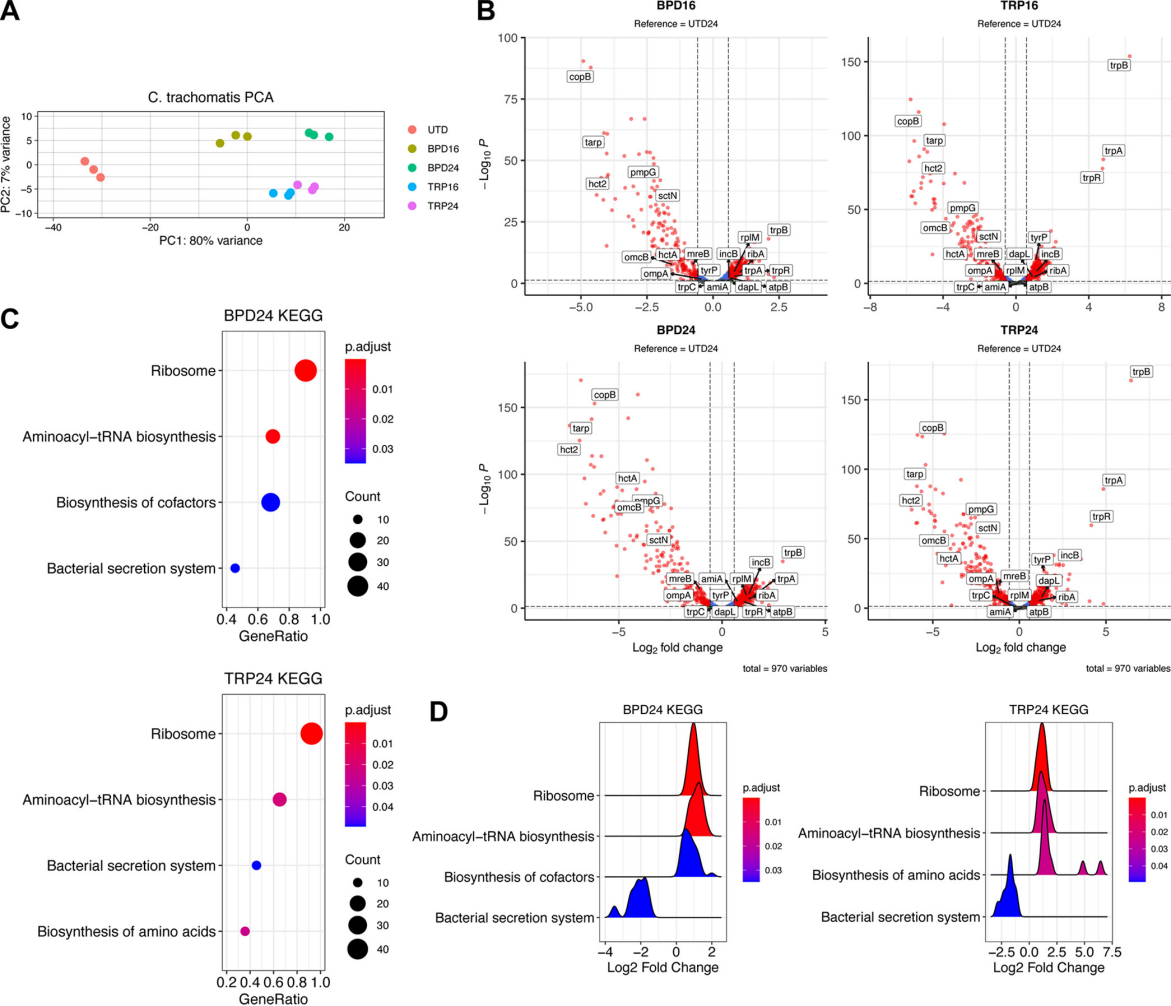
The persistent chlamydial transcriptome is broadly conserved across different nutritional conditions. (A) Principal-component analysis (PCA) of the chlamydial transcriptomes derived from the various nutrient-deprived conditions. All data are from 3 independent biological replicates (N = 3). (B) Volcano plots of the differentially expressed (DE) genes under each persistence-inducing condition (adjusted *P*-value < 0.05, |Fold change| > 1.5). Red, DE, blue, *P < *0.05, |FC| < 1.5, green, *P > *0.05, |FC| > 1.5, gray, *P > *0.05, |FC| < 1.5. (C) Dot plots for enriched pathways identified by clusterProfiler in BPD24 and TRP24. Dot size reflects the number of genes enriched in the pathway and dot color indicates the statistical significance of pathway enrichment. (D) Ridge plots for enriched pathways identified by clusterProfiler in BPD24 and TRP24. Ridges represent normalized density of genes plotted against their Log_2_ fold change. Ridge color reflects statistical significance of pathway enrichment.

Our phenotypic analyses indicated a high degree of concordance in the establishment of chlamydial persistence by different nutritional stressors. Whereas most bacteria would elicit unique transcriptional responses to distinct nutritional insults, we reasoned that chlamydial persistence is likely distinguished by a conserved transcriptional program. Indeed, analysis of our RNA-seq data sets revealed that the transcriptional response of *Ctr* is largely conserved across conditions. Complete details of the differential expression analysis can be found in Data set S1. Principal-component analysis of the variation between chlamydial transcriptomes demonstrated clustering of all treated conditions (Fig. 4A). This indicated that the chlamydial transcriptome associated with different stressors and treatment regimens is highly similar. This is further emphasized by surveying the landscape of statistically significant DE genes (adjusted *P*-value < 0.05, |FC| > 1.5), where we observe all conditions produce a more significantly downregulated than upregulated set of genes (Fig. 4B). Notably, we observe consistently strong downregulation of virulence-associated genes such as the polymorphic outer membrane protein, *pmpG* (28, 29), and the histone-like, nucleoid condensing genes *hctA* and *hctB* (*hct2*) (12, 30, 31). Furthermore, we note that RNA-seq reproduces the substantial upregulation of *trpRBA* in TRP as observed by RT-qPCR (Fig. 3D). Thus, our RNA-seq data recapitulates expected trends based on previous gene expression studies in *Ctr.*

Next, we subjected the sets of DE genes from each condition to the GSEA pipeline as above for the HeLa transcriptomes. Due to the gross similarity between treatment regimens, we limit the analysis here to BPD24 and TRP24 but provide complete details of the analysis for each condition in Data set S2. Among the significantly enriched pathways identified, we observed a high level of agreement between treatments, with the pathways “Ribosome” (ctb03010) and “Aminoacyl-tRNA biosynthesis” (ctb00970) significantly enriched in all conditions, and “Bacterial secretion system” (ctb03070) significantly enriched in 3 of 4 conditions (Fig. 4C). Consistent with previous studies, “Ribosome” and “Aminoacyl-tRNA biosynthesis” pathways are activated, while the virulence-associated “Bacterial secretion system” pathway is suppressed (Fig. 4D) (12). Interestingly, GSEA also revealed the unique enrichment of categories specific to a given treatment. BPD24 significantly activates the “Biosynthesis of cofactors” (ctb01240) pathway, whereas TRP24 results in significant enrichment of the “Biosynthesis of amino acids” (ctb01230) pathway (Fig. 4C and D). While the enrichment of “Biosynthesis of amino acids” is likely driven by the substantial upregulation of the *trpRBA* operon in TRP24, the identification of functionally unique pathways under both nutrient conditions implies that while the overarching transcriptome may be similar, *Ctr* possess a limited ability to tailor their transcriptional response to a given stress.

### The “core” persistent transcriptome is decorated by differentially expressed “accessory” genes unique to each nutritional condition.

**FIG 5:**
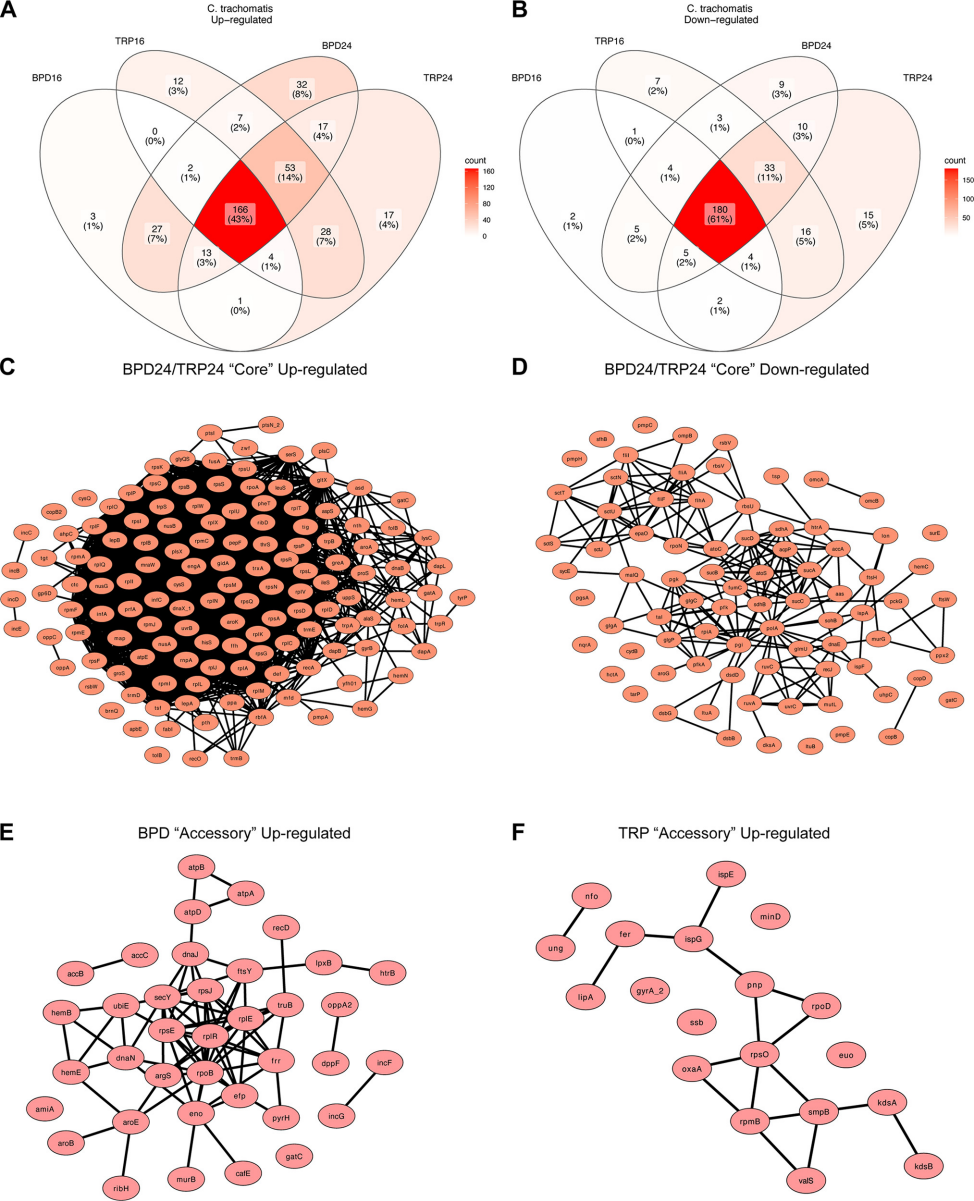
The core persistent chlamydial transcriptome is decorated by unique accessory genes in each nutritional condition. (A) Venn diagram of all differentially upregulated genes across the various nutritional conditions. Color shading reflects the relative number of genes in each cross-section of the Venn diagram. (B) Same as in (A) for all differentially downregulated genes. (C) Network plot of the core upregulated genes in BPD24 and TRP24. Network consists of those genes that were recognized by the STRING-db server. (D) Same as in (C) for the core downregulated genes. (E) Network plot of the upregulated accessory genes across BPD conditions. Network consists of those genes recognized by the STRING-db server. (F) Same as in (E) for the TRP conditions.

Characterization of the chlamydial transcriptome across multiple nutritional conditions revealed a conserved, “core” persistent transcriptome in *Ctr.* This core persistent transcriptome consisted of 43% of the upregulated genes (Fig. 5A) and 61% of the downregulated genes across conditions (Fig. 5B). Network maps were generated from the core DE up- or downregulated genes obtained from BPD24 and TRP24 (Fig. 5C and D). The full list of core DE genes can be found in Data set S3. Within the upregulated set of genes, a highly interconnected node emerged representing several ribosomal subunit and translation adaptor genes (Fig. 5C), consistent with previous reports on the persistent chlamydial transcriptome (12). However, it is unclear if this upregulation leads to increased translational activity (32). Rather, this may reflect a preparation for reactivation such that translation can rapidly resume once conditions improve. In the downregulated set of core genes, a more disconnected network was produced, though distinct nodes emerged, including genes related to central metabolic functions like glycolysis, the tricarboxyclic acid cycle and oxidative phosphorylation (e.g., *sucD*, *sdhA*, *pfk*, *etc.*) and type III secretion system components (e.g., *sctN*, *sctT*, *sctU*) (Fig. 5D). In addition, the core set of genes included several developmentally late genes (*omcB*, *hctA*, *omcA*, *etc.*) and virulence genes (*tarp*, *copB*, *pmpE*, *etc.*), though these generally had very few network connections. These data collectively confirm previous findings on chlamydial persistence, but provide a more detailed view of the conserved transcriptional stress response in *Ctr.*

10.1128/mbio.02719-22.8 DATA SET S3 Lists of chlamydial core and accessory DE genes during nutrient starvation. Download Data Set S3, XLSX file, 0.1 MB. Copyright © 2022 Pokorzynski et al. 2022 Pokorzynski et al. https://creativecommons.org/licenses/by/4.0/ This content is distributed under the terms of the Creative Commons Attribution 4.0 International license.

Despite the high degree of transcriptional conservation, *Ctr* maintained many DE “accessory” genes that were treatment-specific (Fig. 5A and B). Interestingly, a higher number of the upregulated genes were unique, with 32 and 17 of all upregulated genes being unique to BPD24 and TRP24, respectively (Fig. 5A), compared to only 9 and 15 uniquely downregulated genes for the respective conditions (Fig. 4B). Notably, fewer genes were uniquely upregulated in the 16 h treatment conditions (Fig. 5A), implying that the persistent transcriptome is an active and progressive chlamydial stress response. Network plots were generated for the uniquely upregulated genes for BPD and TRP (Fig. 5E and F). The complete list of up- and downregulated accessory genes can be found in Data set S3. Among the recognized genes passed to the String database, the BPD accessory transcriptome contained many genes related to translation (*rpsJ*, *rplR*, *efp*, *etc.*), cofactor biosynthesis (*hemB*, *accB*, *aroE*, *etc.*) and energy transduction through ATP synthase (*atpA*, *atpB*, *atpD*) (Fig. 5E). Intriguingly, 2 genes unique to BPD, *incG* and *incF*, encode inclusion membrane proteins involved in manipulating host subcellular processes (33, 34). For the TRP accessory transcriptome, a smaller translation-related node emerged (*rpsO*, *rpmB*, *smpB*, *etc.*) along with a few pairs of related genes, such as *ispE* and *ispG*, involved in the non-mevalonate methylerythritol phosphate pathway of isoprenoid biosynthesis (Fig. 5F).

To validate the accessory transcriptome, we assayed differential transcription of several genes by RT-qPCR. For BPD, we selected *amiA*, encoding a peptidoglycan (PG) amidase, and *incG* and *incF*, which encode inclusion membrane proteins as noted above. For TRP, we selected the superoxide dismutase-encoding gene, *sodM*, and *ispE*, encoding a 4-diphosphocytidyl-2-C-methyl-d-erythritol kinase that has previously been implicated in nucleoid condensation of the chlamydial EB (35). We found that *amiA*, *incG*, and *incF* were all uniquely upregulated in BPD24 relative to all other conditions (Fig. S2). Notably, the unique upregulation of *amiA* transcription is consistent with the lack of PG at the division septum in iron-starved Chlamydia (36). The transcription of *sodM* was more strongly upregulated at TRP16 than TRP24, consistent with the RNA-seq (Data set S1), but we also found transcription of *sodM* to be significantly upregulated in BPD24 (Fig. S2). This is also apparent from the RNA-seq differential expression analysis, though the degree of *sodM* upregulation in BPD24 did not pass our fold change cutoff (1.5-fold; Data set S1). Finally, the transcription of *ispE* was not significantly different under any condition compared to UTD24, but was on average 1.6-fold higher in TRP relative to BPD, consistent with the RNA-seq (Fig. S2 and Data set S1). As a control, we also validated one gene that was upregulated in the core transcriptome, *tyrP* (*tyrP_1*), encoding an aromatic amino acid transporter, which has previously been described to not respond to tryptophan starvation (37). As expected, based on the RNA-seq data, *tyrP* expression was highest in the TRP conditions, and also significantly upregulated by BPD, confirming it as a member of the core persistent transcriptome (Fig. S2). In total, these data imply that the persistent chlamydial transcriptome is highly conserved across conditions but retains the ability to deploy stress-specific, and possibly adaptive, responses.

10.1128/mbio.02719-22.2 FIG S2 Validation of differential expression for selected accessory genes during nutritional stress. All plots represent the mean and standard deviation of 3 independent biological replicates (N = 3). Statistical significance in all panels was determined by one-way ANOVA followed by Tukey’s post-hoc test of honestly significant differences (two-tailed). *, *P < *0.05, **, *P < *0.01, ***, *P < *0.001, ns, not significant. Download FIG S2, PDF file, 0.2 MB. Copyright © 2022 Pokorzynski et al. 2022 Pokorzynski et al. https://creativecommons.org/licenses/by/4.0/ This content is distributed under the terms of the Creative Commons Attribution 4.0 International license.

### Suppression of host cell purine metabolism is reflected by transcriptional activation of GTP-dependent cofactor biosynthesis pathways in persistent chlamydiae.

**FIG 6:**
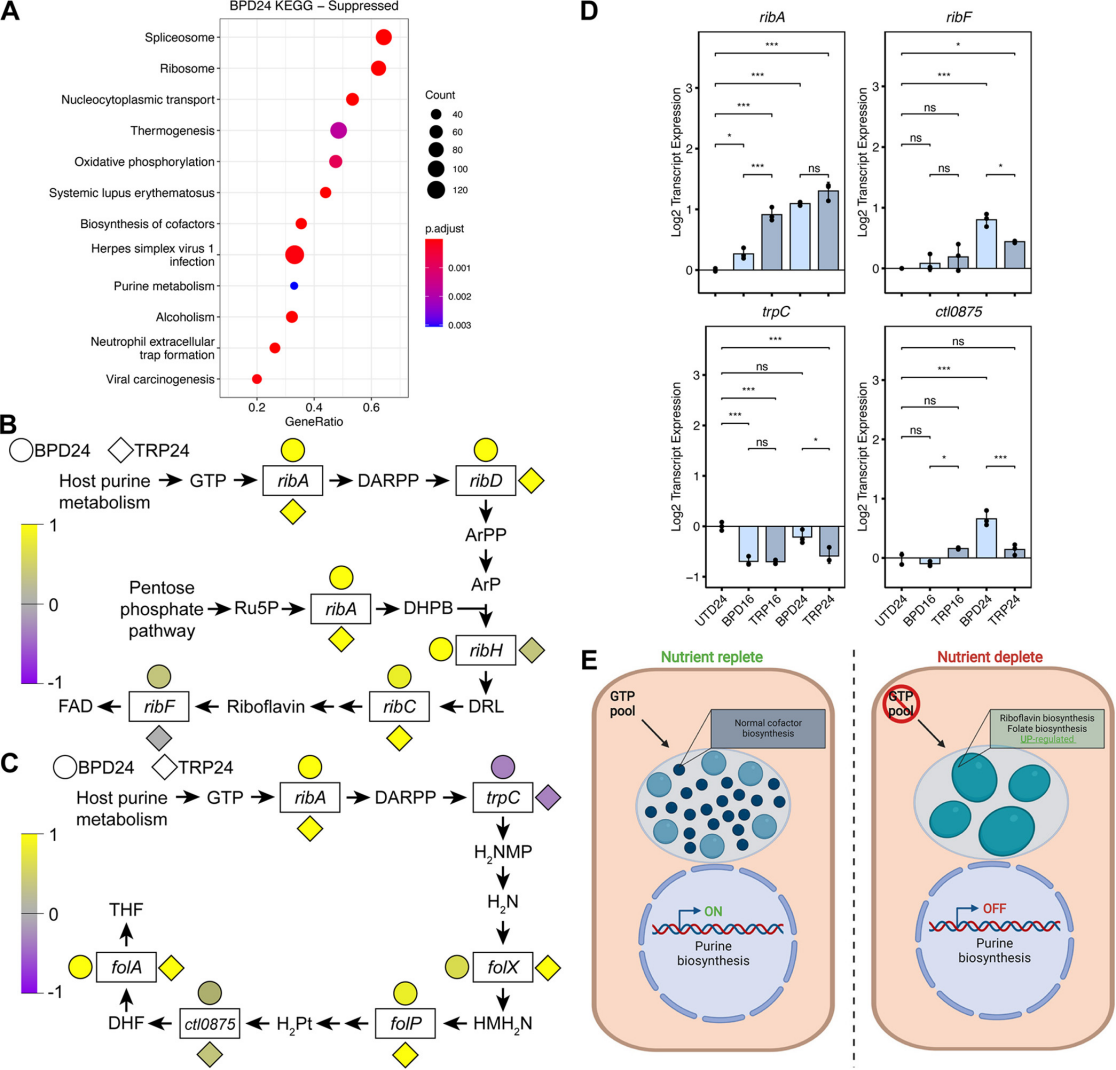
Suppressed host cell purine metabolism is reflected by an upregulation of GTP-dependent biosynthetic pathways in Chlamydia trachomatis. (A) Dot plot for the 10 most activated or suppressed pathways identified by clusterProfiler in BPD24. Dot size reflects the number of genes enriched in the pathway and dot color indicates the statistical significance of pathway enrichment. (B) Simplified depiction of the chlamydial riboflavin biosynthetic pathway with color-coded normalized gene expression data for each gene represented by adjacent circles (BPD24) or diamonds (TRP24). DARPP = 2,5-diamino-6-(5-phospho-d-ribosylamino)-pyrimidin-4(3H)-one, ArPP = 5-amino-6-(5-phospho-d-ribosylamino)uracil, ArP = 5-amino-6-(5-phospho-d-ribitylamino)uracil, Ru5P = ribulose-5-phosphate, DHPB = 3,4-dihydroxy-2-butanone 4-phosphate, DRL = 6,7-dimethyl-8-ribityllumazine, FAD = flavin adenine dinucleotide. (C) Same as in (B) for the chlamydial tetrahydrofolate biosynthetic pathway. DARPP = 2,5-diamino-6-(5-phospho-d-ribosylamino)-pyrimidin-4(3H)-one, H_2_NMP = 7,8-dihydroneopterin 3-phosphate, H_2_N = 7,8-dihydroneopterin, HMH_2_N = 6-hydroxymethyl-7,8-dihydropterin, H_2_Pt = 7,8-dihydro-pteroate, DHF = 7,8-dihydrofolate, THF = 5,6,7,8-tetrahydrofolate. (D) Gene expression profiles of selected differentially regulated genes in the riboflavin and THF biosynthetic pathways under original persistence models. Statistical significance in all panels was determined by one-way ANOVA followed by Tukey’s *post hoc* test of honestly significant differences (two-tailed). *, *P < *0.05, **, *P < *0.01, ***, *P < *0.001, ns, not significant. (E) Proposed model of reciprocal upregulation of chlamydial riboflavin and THF biosynthesis in the presence of the nutrient-starved suppression of purine metabolism. Created with BioRender.com.

The contrast between the distinct transcriptional profiles of the infected host cell and the conserved persistent chlamydial transcriptomes prompted further investigation into the relevance of host pathways downstream of the primary (i.e., iron or tryptophan starvation) stresses. We extended our analysis of the HeLa GSEA data to include significantly suppressed pathways, which also differed between TRP and BPD. Suppressed pathways uniquely associated with BPD24 (Fig. 6A and Data set S2), included “Purine metabolism” (hsa00230), which was intriguing given that *Ctr* is auxotrophic for GTP (38, 39). In addition to DNA replication, transcription, and translation, GTP is also an essential substrate for chlamydial riboflavin and tetrahydrofolate (THF) biosynthesis (40). Because these 2 pathways fall within the broader “Biosynthesis of cofactors” pathway identified as significantly enriched by *Ctr* in BPD24 (Fig. 4C and D), we hypothesized that this enrichment is linked to the suppression of host GTP biosynthesis. We extracted the expression data for both the host and chlamydial pathways from the RNA-seq data sets for the BPD24 and TRP24 conditions and observed that host purine metabolism was markedly downregulated transcriptionally in BPD24 compared to TRP24, particularly along the biosynthetic arm leading from ribose-5-phosphate to IMP (Fig. S3A and B). However, the gene IMPDH1, encoding the rate-limiting enzyme IMP dehydrogenase, was transcriptionally downregulated in both BPD24 and TRP24, indicating a possible convergence between conditions in the suppression of GTP biosynthesis.

10.1128/mbio.02719-22.3 FIG S3 Differential expression of genes in the purine metabolism pathway of persistently infected HeLa cells. (A) Pathway data for BPD24 extracted by Pathview. (B) Pathway data for TRP24 extracted by Pathview. Download FIG S3, PDF file, 0.5 MB. Copyright © 2022 Pokorzynski et al. 2022 Pokorzynski et al. https://creativecommons.org/licenses/by/4.0/ This content is distributed under the terms of the Creative Commons Attribution 4.0 International license.

In Fig. 6B and Fig. 6C, we depict the chlamydial riboflavin and THF biosynthetic pathways, simplified for clarity, with color-coded expression data for each gene annotated in the pathway. We then qualitatively compared the normalized expression of the two pathways between BPD24 and TRP24 to determine their correlation with the pathway enrichment results. In contrast to the unique enrichment of pathways between conditions, genes within the riboflavin and THF biosynthetic pathways are broadly upregulated in both BPD24 and TRP24, though differences exist in key genes. For example, the gene *ribF*, encoding the bifunctional riboflavin kinase/FMN adenylyltransferase which catalyzes the final step of flavin adenine dinucleotide (FAD) biosynthesis, appears upregulated in BPD24 relative to TRP24 (Fig. 6B).

To validate the transcriptional regulation of the chlamydial riboflavin and THF pathways identified by RNA-seq, we assayed the expression of a subset of genes from these pathways under our various nutrient-depleted conditions by RT-qPCR (Fig. 6D), focusing on those genes that either appeared differentially regulated between BPD24 and TRP24 (e.g., *ribF*) (Fig. 6B), or that encoded enzymes which catalyze important steps in these pathways, such as the GTP cyclohydrolase RibA, the promiscuous enzyme TrpC which shunts the product of the RibA toward THF biosynthesis (40), and the gene *ctl0875* (*ct611*, *folC2*) which encodes a nonorthologous, alternate folylglutamate synthase (40, 41). Relative to UTD24, the transcription of *ribA* was significantly upregulated in BPD24 and TRP24, but not statistically distinguishable between these groups (Fig. 6D). In contrast, *ribF* exhibited significantly higher expression in BPD24 compared to TRP24, confirming the specific transcriptional regulation of this gene between conditions. Both *trpC* and *ctl0875* were also more strongly upregulated in BPD24 compared to TRP conditions, but generally maintained profiles consistent with the RNA-seq (that is, downregulation for *trpC* and marginal upregulation for *ctl0875*.) In accordance with the overall transcriptional profile of persistent chlamydiae in different nutritional conditions being characterized by a core and accessory component, we find here that this can extend even to individual pathways. Nevertheless, the overall similarity in expression of the chlamydial riboflavin and THF biosynthetic pathways indicated that in both conditions *Ctr* responds to a similar stimulus. We therefore hypothesized that the reduction of GTP levels could be “sensed” by *Ctr*, yielding increased expression of genes involved in the GTP-requiring riboflavin and tetrahydrofolate biosynthetic pathways (Fig. 6E).

### GTP depletion induces chlamydial persistence and is a common metabolic consequence of nutritional stress in epithelial cells.

**FIG 7:**
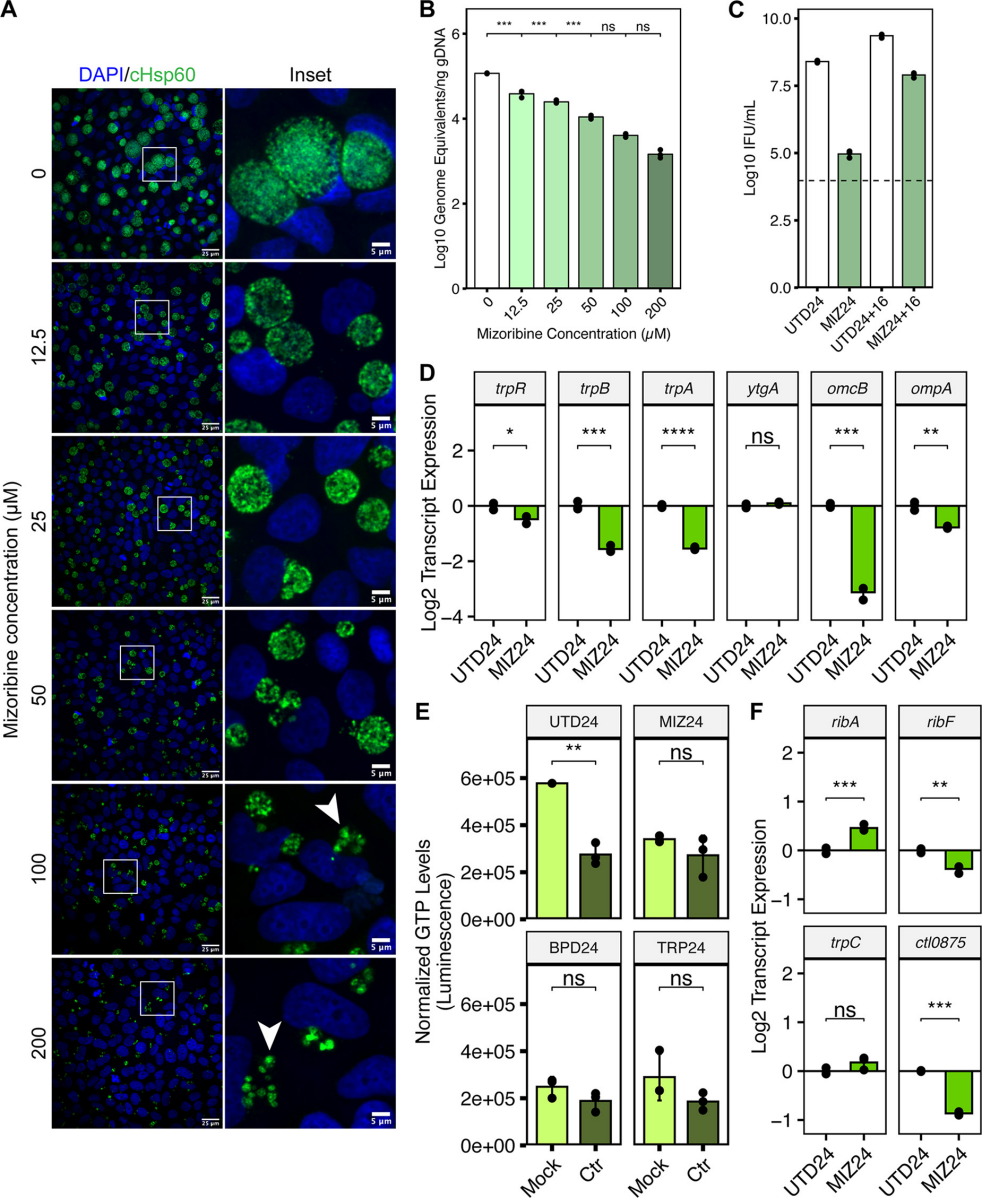
Inhibition of GTP biosynthesis is sufficient to induce chlamydial persistence and phenocopies the reduction in host GTP levels and the transcriptional regulation of cofactor biosynthesis by Chlamydia. (A) Immunofluorescent confocal microscopic analysis of chlamydial morphology following a 2-fold dilution series of mizoribine (MIZ) beginning at the time of infection. Micrographs are representative of at least 3 independent biological replicates (N = 3). Chlamydial organisms were detected by immunostaining against the cytosolic Hsp60 homologs, GroEL_1-GroEL_3. Nuclei were detected by staining with DAPI. Arrowheads indicate aberrantly enlarged bacteria. (B) Determination of genome equivalents under the same MIZ dilution series in (A) by quantitative PCR against the *euo* locus. (C) Measurement of infectious progeny generation during 100 μM MIZ (MIZ24) in a reinfection assay. Reactivation was allowed to proceed for 16h by replacement of MIZ-containing media with fresh media. Dotted line indicates calculated limit of detection for the assay. (D) Gene expression profiles for various nutritionally- or developmentally-regulated chlamydial genes during MIZ24 treatment. Statistical significance in all panels was determined by pairwise two-sided unpaired Welch’s *t* test for unequal variance. *, *P < *0.05, **, *P < *0.01, ***, *P < *0.001. All plots represent the mean and standard deviation of three independent biological replicates (N = 3). (E) Determination of intracellular GTP levels across persistence-inducing conditions using the modified GTPase-Glo assay. All values for each replicate were normalized to the mean of the untreated, mock-infected control group. Statistical significance in all panels was determined by pairwise two-sided unpaired Welch’s *t* test for unequal variance. *, *P < *0.05, **, *P < *0.01, ***, *P < *0.001, ns, not significant. (F) Gene expression profiles of selected differentially regulated genes in the riboflavin and THF biosynthetic pathways under MIZ24 condition. Statistical significance in all panels was determined by one-way ANOVA followed by Tukey’s *post hoc* test of honestly significant differences (two-tailed). *, *P < *0.05, **, *P < *0.01, ***, *P < *0.001, ns, not significant.

Validation of our model that *Ctr* could sense and respond to GTP deprivation required an independent means of specifically depleting the host GTP pool. We, therefore, turned to mizoribine (MIZ), a selective inhibitor of IMP dehydrogenase (IMPDH1/2) and GMP synthase (GMPS) (42, 43). MIZ is a potent inhibitor of mammalian GTP biosynthesis (44) and therefore provided a suitable tool to evaluate whether GTP starvation alone influenced chlamydial development and gene regulation. We treated *Ctr*-infected HeLa cells with a 2-fold dilution series of MIZ for 24 h, starting at the time of infection. Chlamydial morphology and genome replication were acutely sensitive to MIZ, with a perceptible reduction in inclusion size (Fig. 7A) and significant decrease in genome copy number (Fig. 7B) detectable at the lowest concentration of MIZ tested, 12.5 μM. However, we sought to determine whether MIZ treatment could induce a persistent state in *Ctr.* We observed that by 100 μM MIZ treatment, abnormal chlamydiae could be detected by confocal immunofluorescent microscopy (Fig. 7A) and furthermore that genome equivalents at this concentration were not statistically distinguishable from either the 50 or 200 μM treatments (Fig. 7B), suggesting that genome replication had been stalled. Therefore, we moved forward with the 100 μM MIZ treatment (hereafter MIZ24) and determined if *Ctr* retained viability when a reactivation (i.e., withdrawal of the inhibitor) period of 16 h was introduced (Fig. 7C). Recoverable IFUs were reduced roughly 2700-fold in MIZ24 relative to UTD24 but recovered to a level only 3-fold lower than UTD24 after reactivation. Thus, *Ctr* adopts an aberrant morphology and stalls genome replication in the presence of MIZ but retains viability, which are physiological hallmarks of chlamydial persistence.

We next returned to the panel of genes analyzed in Fig. 3D to compare the gene expression profile of MIZ24 to other well characterized persistence models. Unlike BPD and TRP, MIZ downregulated the expression of the *trpRBA* operon, but reproduced the transcriptional profile of *ytgA*, *omcB*, and *ompA* found under established persistence models (Fig. 3D and Fig. 7D). It is not surprising that *trpRBA* gene expression profiles were not replicated under this condition because the nutritional co-repressors (i.e., iron and tryptophan) were not expected to be negatively affected by MIZ. We additionally analyzed expression of the curated panel of accessory genes (Fig. S2) in the MIZ24 condition and found that persistence induced by MIZ exhibited transcriptional signatures reminiscent of BPD24, including significant upregulation of *amiA*, *incG*, and *incF*, while *sodM* and *ispE* were not statistically distinguishable from UTD24 (Fig. S4). We note that *ispE* transcription in MIZ24 still appears downregulated, similar to its expression in BPD24. The transcription of *tyrP* was marginally but significantly upregulated, again consistent with it being a part of the core persistent transcriptome.

10.1128/mbio.02719-22.4 FIG S4 Analysis of accessory gene expression in MIZ24. Statistical significance in all panels was determined by pairwise two-sided unpaired Welch’s *t*-test for unequal variance. *, *P < *0.05, **, *P < *0.01, ***, *P < *0.001. All plots represent the mean and standard deviation of three independent biological replicates (N = 3). Download FIG S4, PDF file, 0.01 MB. Copyright © 2022 Pokorzynski et al. 2022 Pokorzynski et al. https://creativecommons.org/licenses/by/4.0/ This content is distributed under the terms of the Creative Commons Attribution 4.0 International license.

With the validation of MIZ as a tool for starving *Ctr* of GTP and inducing persistence, we next assayed intracellular GTP levels in mock-infected or *Ctr-*infected HeLa cells during nutritional stress. To measure the host GTP pool, we adapted a commercially available kit for assaying GTPase activity (see Materials and Methods). By comparing mock-infected samples, we observed that all nutrient-depleted conditions resulted in a reduction of GTP levels comparable to that observed with MIZ24 (Fig. 7E). Interestingly, we observed that infection alone decreased intracellular GTP levels, which may reflect increased competition for this nucleotide between host and pathogen. However, infection could not further reduce the level of GTP from any nutrient-starved condition, suggesting that GTP was inaccessible to *Ctr*. Whether this is solely the effect of suppressed purine metabolism or if GTP sequestration or depletion occurs is unknown. Therefore, the host cell responds to nutrient limitation by depleting GTP pools, which negatively impacts *Ctr* as they compete for this critical nutrient to sustain replication and development. We note however that only BPD24 was able to reduce total luminescent output from this assay, which reflects the gross suppression of purine metabolism identified by pathway-level analysis (Fig. S5 and Fig. 6A).

10.1128/mbio.02719-22.5 FIG S5 Total luminescent output in unconverted ([ATP]) and converted ([GTP]+[ATP]) reactions for the measurement of intracellular GTP levels. Note that the values in ([GTP]+[ATP]) samples are higher than those in [ATP] alone, reflecting the conversion of the GTP pool. All plots represent the mean and standard deviation of three independent biological replicates (N = 3). Statistical significance in all panels was determined by one-way ANOVA followed by Tukey’s post-hoc test of honestly significant differences (two-tailed). *, *P < *0.05, **, *P < *0.01, ***, *P < *0.001, ns, not significant. Download FIG S5, PDF file, 0.2 MB. Copyright © 2022 Pokorzynski et al. 2022 Pokorzynski et al. https://creativecommons.org/licenses/by/4.0/ This content is distributed under the terms of the Creative Commons Attribution 4.0 International license.

Finally, we assayed the expression of the same subset of genes from the chlamydial riboflavin and THF biosynthetic pathways in MIZ24 to determine whether *Ctr* responded similarly at the transcriptional level to direct GTP starvation (Fig. 7F). In comparison with BPD24 and TRP24, we find that MIZ24 significantly increased expression of *ribA* and did not alter *trpC* expression, more closely resembling the BPD24 condition. However, unlike BPD24, both *ribF* and *ctl0875* were significantly downregulated by MIZ24, indicating additional regulatory inputs during iron starvation that modulate the expression of these pathways. This finding also implied that GTP starvation, while sufficient in the context of mizoribine treatment, is not the major mechanism by which iron starvation induces persistence. Rather, GTP depletion is one of many contributors to persistence.

### Inhibition of IMPDH acts synergistically with iron starvation to negatively regulate chlamydial growth.

**FIG 8:**
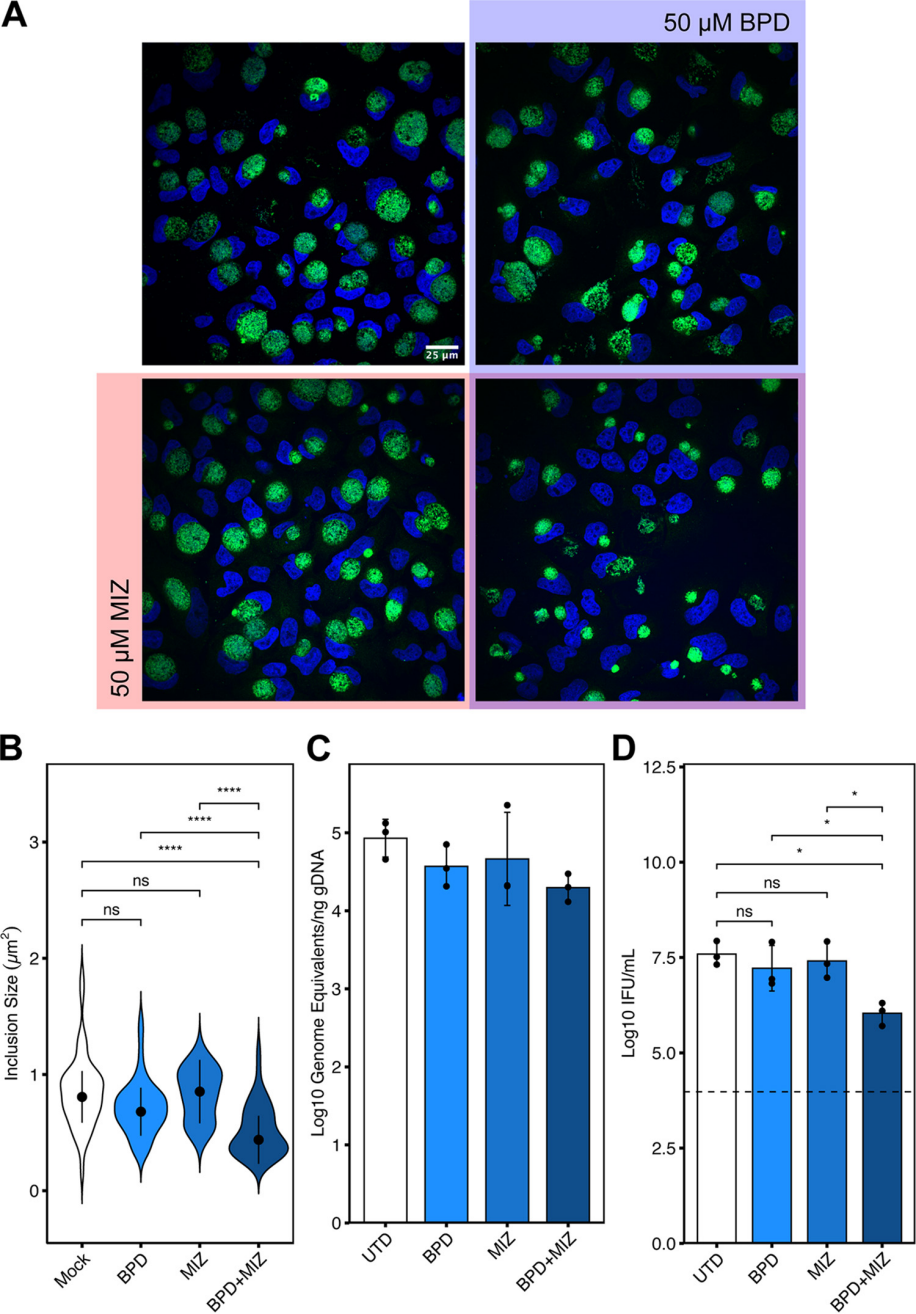
Combined treatment of subinhibitory concentrations of mizoribine and bipyridyl reduces chlamydial inclusion size synergistically. (A) Representative immunofluorescent confocal micrographs of *Ctr*-infected HeLa cells treated at 8 hpi with either 50 μM BPD (blue), 50 μM MIZ (Red), both MIZ and BPD (purple overlap) or left untreated (uncolored quadrant). Cells were fixed at 24 hpi (16h treatment regimen) and chlamydial inclusions were detected by staining with anti-cHsp60 antibodies. Nuclei were visualized by DAPI staining. (B) Quantification of inclusion size determined from data collected in panel (A). Width of violin pots represent the statistical density of observed inclusion sizes. The black dots represent the median of the data, and the black bars indicate the median absolute deviation. Statistical significance was determined by a One-Way Kruskal-Wallis test and *post hoc* Wilcoxon rank sum test with Bonferroni’s correction for multiple comparisons. *, *P < *0.05, **, *P < *0.01, ***, *P < *0.001, ns, not significant. The measurement of a single inclusion was considered one observation and N = 45 for each sample, which was derived from random, equal sampling of the entire data set collected from three independent biological replicates. (C) Analysis of genome equivalents by qPCR of the *euo* locus under the indicated conditions as described above. (D) Measurement of infectious progeny generation under the indicated conditions as described above. Statistical significance in panel (D) was determined by one-way ANOVA followed by Tukey’s *post hoc* test of honestly significant differences (two-tailed). *, *P < *0.05, **, *P < *0.01, ***, *P < *0.001, ns, not significant.

For downregulated purine biosynthesis to be relevant to the development of chlamydial persistence induced by unrelated stressors, we reasoned that it must act in concert with stimuli such as iron starvation to impart a defect on chlamydial growth and development. To directly test this hypothesis, we treated *Ctr*-infected HeLa cells with subinhibitory concentrations of MIZ or BPD under our less severe treatment regimen of 8 hpi + 16 h treatment (Fig. 2A and Table 1). In theory, similar experiments could be performed under subinhibitory tryptophan starvation protocols, but to maintain the same treatment regimen this would require defining a relevant minimal tryptophan concentration in a cell culture model of infection, which is not straight-forward. Therefore, we assayed chlamydial inclusion size when exposed to 50 μM MIZ or BPD either alone or in combination (Fig. 8A and B). Qualitative assessment of inclusions size by immunofluorescent confocal microscopy indicated that neither treatment alone was sufficient to substantially reduce chlamydial inclusion size (Fig. 8A), and this observation was confirmed by quantification (Fig. 8B). In contrast, the combined treatment of subinhibitory concentrations of MIZ and BPD produced a significant, synergistic defect in chlamydial inclusion size that was apparent both qualitatively and quantitatively (Fig. 8A and B). To determine if the synergistic decrease in inclusion size following BPD and MIZ co-treatment corresponded to defects in chlamydial growth and development, we assayed both genome equivalents and infectious progeny generation (Fig. 8C and D). A marginal synergistic defect of BPD and MIZ co-treatment was observed on chlamydial genome equivalents (Fig. 8C), while a statistically significant, synergistic defect was observed on infectious progeny generation following co-treatment (Fig. 8D). Decreased infectious progeny and chlamydial genome replication are both consistent with the development of persistence only in the presence of both nutritional stressors. Together, these data suggest that downregulated IMPDH transcription during iron or tryptophan starvation likely exacerbates the primary stress and contributes to the development of persistence.

While recapitulating this phenomenon by directly manipulating IMPDH transcript levels would be ideal, this would require knowledge of the specific transcript-to-protein ratio in infected, stressed host cells, and furthermore would require knowledge of the specific activity of IMPDH under these conditions. Given the established relationship between stress and altered IMPDH activity (45, 46) and the observation that pharmacological inhibition of IMPDH activity reproduces the persistent phenotype (Fig. 7), we cannot conclude that the transcriptional defect alone explains this phenomenology. Rather, it is likely an important signature that indicates a broader regulation taking place which negatively influences chlamydial development during nutritional or immunological stress.

## DISCUSSION

Historically, models of nutritional stress during infection have operated under the assumption that downstream phenotypic consequences to the pathogen can be directly attributed to the depletion of the nutrient in question. We challenge this assumption by demonstrating that the nutrient-deprived host cell can deploy an unrelated antibacterial nutritional stress by suppressing GTP biosynthesis, essentially amplifying and diversifying the stress acting upon the pathogen. How this is accomplished is presently unclear, though we show here that transcriptional suppression of purine biosynthesis likely plays an important role, particularly during iron starvation. Alternatively, it could be argued that chelation of iron by BPD disrupts the iron-sulfur cluster-containing amidophosphoribosyltransferase, PPAT, which converts ribose-5-phosphate to ribosylamine-5-phosphate (47). However, this biochemical explanation does not account for the broader transcriptional downregulation of purine metabolism during BPD24 treatment or the reduction of GTP levels in the TRP24 condition. Thus, a more fundamental process seems to be at play – one that may ultimately benefit the host during persistent infection. In agreement with this is the observation that distantly related eukaryotes, such as the budding yeast Saccharomyces cerevisiae, also suppress purine biosynthesis in the absence of iron, despite lacking iron-dependent enzymes in these pathways (48, 49). Downregulation of purine biosynthesis could be a de-prioritization of host DNA replication and translation in response to depletion of iron or the essential amino acid tryptophan, making this a host stress adaptation strategy with concomitant antibacterial benefit. A broader implication is that a host cell under stress may not necessarily be compromised in dealing with an intracellular pathogen.

The pro-inflammatory cytokine IFNγ has been attributed a major role in the host anti-chlamydial immune response (50). Much of this anti-chlamydial activity has been explained by the IFNγ-mediated induction of IDO1, which catabolizes host cell tryptophan pools to starve *Ctr* of this essential amino acid (14, 51). Whether IDO1 overexpression is sufficient to inhibit chlamydial growth has not been investigated. Instead, IDO1 relevance is based on tryptophan supplementation studies and subsequent rescue of normal growth (7, 14). Similarly, studies with IDO1 inhibitors, such as levo-1-methyl-tryptophan (L-1MT) only led to a partial rescue of growth (52). Both published results remain consistent with additional stresses distinct from tryptophan catabolism by IDO1 being involved in IFNγ-mediated growth inhibition. IFNγ has also been shown to suppress purine metabolism by inhibiting eIF4E expression to reduce translation of genes in the purine biosynthetic pathway in primary human macrophages (an effect that depends on IFNγ-mediated IDO1 induction) (53, 54). This would be analogous to GTP depletion being an outcome of tryptophan starvation. Thus, it may be that reducing purine nucleotide levels, and specifically the GTP pool, is an evolved and redundant immune response of the host cell.

Notably, our work is not the first to implicate purine biosynthesis, and IMPDH1/2 specifically, in the intracellular growth of *Ctr* (55). Using a genome-wide RNAi screen, Rother et al. identified IMPDH as a key regulator of chlamydial growth under normal conditions, demonstrating growth inhibition by independent knock-down of IMPDH expression and pharmacological inhibition of IMPDH by the compound mycophenolate mofetil (MMF). The growth defect induced by MMF treatment was at least partially rescued by supplementation of the infected-cell culture with GMP. Moreover, the authors demonstrated that MMF treatment was sufficient to reduce bacterial burden and pathology *in vivo.* From these data, the authors concluded that IMPDH may be a viable therapeutic target against chlamydial infections. We report here that while IMPDH is clearly important for chlamydial growth and development, its inhibition leads to persistence rather than bacterial killing, a distinction that was not directly addressed by Rother et al. This information is essential given the ramifications of persistence on pathology and success of antibiotic regimen (20, 56, 57).

The characterization of unique, “accessory” components of the persistent chlamydial transcriptome is an important advancement. Not only does this underscore the ability of bacteria with evolutionarily reduced genomes to retain condition-specific transcriptional regulation, but it points to interesting and distinct mechanisms that contribute to the broader chlamydial stress response. The current understanding of chlamydial development, and particularly differentiation, is incomplete insofar as it cannot pinpoint specific molecular cues that promote differentiation. One contributor to differentiation is the metabolite 4-diphosphocytidyl-2-C-methyl-d-erythritol 2-phosphate, generated by the enzyme IspE, which antagonizes the histone-like protein HctA and causes nucleoid decondensation (35). Thus, isoprenoid biosynthesis appears intimately connected to differentiation, but the signals regulating this pathway are unknown. The observation that transcription of the *ispE* gene is upregulated in the tryptophan-starved accessory transcriptome (Fig. 5F and Fig. S2) implies that tryptophan availability may be a relevant signal. Another process that impacts chlamydial development is the manipulation of host subcellular trafficking by the family of inclusion membrane proteins (Incs), through which Chlamydia acquire various nutrients (58). We show here that transcription of the genes *incG* and *incF* is uniquely upregulated in iron-starved and GTP-starved Chlamydia (Fig. 5E, Fig. S2 and Fig. S4). Interestingly, *incG* and *incF* are encoded in the *incDEFG* operon, and *incD* and *incE* were identified as part of the core upregulated persistent transcriptome (Fig. 5C). This suggests a possible condition-specific suppression of *incGF* transcription during tryptophan starvation. Moreover, this raises the possibility that Chlamydia uniquely alter the inclusion membrane proteome during persistence, possibly to acquire crucial host-derived nutrients to retain viability. These findings may provide clues as to relevant intracellular signals regulating chlamydial development.

Collectively, we provide evidence that both primary and secondary effects of a stress contribute to chlamydial persistence. The character of the persistent chlamydial transcriptome also supports the notion that a stressed host cell induces subsequent stresses based on the distinction of a “core” and “accessory” transcriptome. The latter is specific to the original stress, and likely reflects a progressively accumulating and active transcriptional response of the pathogen, as more accessory genes are distinguishable with more severe treatment regimens. On the other hand, the core component could be associated with metabolic consequences that are common to iron and tryptophan starvation, of which reduction in GTP levels is an example. We emphasize that prominent differences remain in the transcriptome components, and a more careful and detailed study is needed to establish their relevance to chlamydial persistence. Based on our combined data, a picture emerges of persistence as a deceptively similar process underpinned by a response that has stress-dependent and -independent components. This response is shaped by the different actions of the primary stress on the pathogen and the host cell, the latter involving the induction of subsequent waves of metabolically oriented stressors that target the pathogen. In other words, despite using a single-stressor experimental model, subsequent stresses with antimicrobial functions are induced, forcing Chlamydia to adapt to not just 1, but 2 or more simultaneous stresses. With a limited repertoire of stress adaptation strategies, *Chlamydiae* are likely more sensitive to these simultaneous stressors than other intracellular bacterial pathogens; this does not discount the potential relevance of such combined effects in these experimental systems but instead argues for their careful examination in future studies.

## MATERIALS AND METHODS

### Cell lines.

Human female cervical epithelial adenocarcinoma HeLa cells (RRID: CVCL_1276) were cultured at 37°C with 5% atmospheric CO2 in Dulbecco’s Modified Eagle Medium (DMEM; Gibco, Thermo Fisher Scientific) supplemented with 10 μg/mL gentamicin, 2 mM l-glutamine, and 10% (vol/vol) filter sterilized fetal bovine serum (FBS). For all experiments, HeLa cells were cultured between passage numbers 3 and 15. HeLa cells were originally authenticated by ATCC via STR profiling and isoenzyme analysis per ATCC specifications.

### Bacterial strains.

Chlamydia trachomatis serovar L2 (434/Bu) was originally obtained from Dr. Ted Hackstadt (Rocky Mountain National Laboratory, NIAID). Chlamydial EBs were isolated from infected HeLa cells at 36 to 40 hpi and purified by density gradient centrifugation essentially as described (59). For infections, at 80 to 90% confluence, HeLa cells were first washed with Hanks Buffered Saline Solution (HBSS; Gibco, Thermo Fisher Scientific) and ice-cold inoculum prepared in HBSS at the indicated multiplicity of infection was overlaid onto the cell monolayer. To synchronize the infection, inoculated cells were then centrifuged for 15 min at 500xRCF, 4°C in an Eppendorf 5810 R tabletop centrifuge with an A-4-81 rotor. The inoculum was then aspirated and pre-warmed DMEM (or relevant media with treatment supplementation) was added to the cells. Infected cultures were then returned to the tissue culture incubator until the indicated time postinfection.

### Treatment conditions.

For iron starvation and media-defined tryptophan starvation, treatment was performed essentially as described previously (23, 24). In brief, 100 mM 2,2-bipyridyl (Sigma-Aldrich; CAS: 366-18-7) prepared in dimethyl sulfoxide (DMSO) was added to complete DMEM (or tryptophan-depleted DMEM-F12, as described below) at a working concentration of 100 μM at the start of infection (BPD24) or at 8 hpi (BPD16). When added after the time of infection, cells were first washed with HBSS prior to bipyridyl treatment. Tryptophan depletion was performed by first washing cells with HBSS and then replacing complete DMEM with tryptophan-depleted DMEM-F12 (U.S. Biological Life Sciences). Media was replaced either at the time of infection (TRP24) or at 8 hpi (TRP16). Treated cells were then returned to the tissue culture incubator for the remainder of the experimental time course. Mizoribine (Sigma-Aldrich, CAS: 50924-49-7) was prepared as a 100 mM stock solution in DMSO, stored at −80°C, and used at the indicated concentrations starting at the time of infection (MIZ24).

### Nucleic acid preparation.

RNA was harvested from C. trachomatis-infected cells by scraping 1 or 2 wells of a 6-well tissue culture plate in a total volume of 500 μL TRIzol Reagent (Thermo Fisher Scientific). Samples were transferred to RNase-free o-ring capped tubes containing ~100 μL volume of zirconia beads and thoroughly vortexed for 10 min to rupture bacterial cells. Zirconia beads were pelleted by centrifugation at 21,000 × *g* for 10 min at 4°C and supernatant was transferred to an RNase-free tube containing 100 μL chloroform (Sigma-Aldrich). Samples were vortexed for 15 s prior to a 10 min room temperature incubation. Phases were then separated by centrifugation at 21,000 × *g* for 15 min at 4°C. The aqueous top layer was transferred to an RNase-free tube containing 250 μL 100% ethanol to precipitate RNA. Samples were briefly vortexed and then applied to an RNA collection column provided in the PureLink RNA minikit (Invitrogen, Thermo Fisher Scientific). RNA was isolated as described by the manufacturer with an on-column DNA digestion using the PureLink DNase Set (Invitrogen, Thermo Fisher Scientific). RNA was eluted in nuclease-free H_2_O and stored at −20°C for short-term storage or −80°C for long-term storage.

cDNA was generated using 1 to 2 μg of RNA as a template for the SuperScript IV Reverse Transcriptase (RT) VILO master mix (Invitrogen, Thermo Fisher Scientific) with a no-RT control reaction in a half-reaction volume following manufacturer protocols. The no-RT control sample was screened for DNA contamination by qPCR against the *euo* locus (see Data set S4 for full list of oligonucleotide primers).

10.1128/mbio.02719-22.9 DATA SET S4 List of oligonucleotide primers used in this study. Download Data Set S4, XLSX file, 0.01 MB. Copyright © 2022 Pokorzynski et al. 2022 Pokorzynski et al. https://creativecommons.org/licenses/by/4.0/ This content is distributed under the terms of the Creative Commons Attribution 4.0 International license.

Genomic DNA (gDNA) was harvested from parallel well(s) of a 6-well plate in 200 μL ice-cold PBS + 10% Proteinase K and processed through the DNeasy blood and tissue kit following manufacture protocols (Qiagen). gDNA was stored at −20°C for short-term storage or −80°C for long-term storage.

For the preparation of RNA-sequencing libraries, 10 μg of RNA collected as described above, with an additional round of on-column DNA digestion, was processed in parallel 5 μg aliquots through the RiboMinus Transcriptome isolation kit (Invitrogen, Thermo Fisher Scientific) essentially as described in the manufacturer protocol with the exception that the magnetic beads were loaded with 3 μL of the pan-prokaryotic rRNA probe as well as 4 μL of the eukaryotic rRNA probe to deplete both host and chlamydial rRNA simultaneously. The resulting rRNA-depleted samples were concentrated in the RNA Clean and Concentrator Kit (Zymo Research) and submitted to the University of Nebraska DNA Sequencing Core for library preparation and RNA-sequencing.

### Library preparation and RNA-sequencing.

Submitted RNA samples were determined to be of suitable quality by fragment analysis on an Agilent 2100 Bioanalyzer (Agilent). TruSeq Stranded Total RNA library preparation kit (Illumina) was used to generate RNA-sequencing libraries following manufacturer protocols with a starting amount of 100 ng rRNA-depleted RNA. Depletion of rRNA in the TruSeq kit was performed by the addition of 2.5 μL each of standard rRNA Removal Mix (RRM) or Prokaryotic RRM. Quality of prepared libraries was determined by concentration and fragment analysis as above. Libraries were sequenced on an Illumina NextSeq NS550 (75 bp single read high output flow cell) or NovaSeq 6000 (75 bp single read SP-100 flow cell). Across replicates, the proportion of sequenced bases with a quality score higher than 30 was at least 95%.

### Host-pathogen RNA-sequencing analysis.

Transcriptomes were processed using the Galaxy server, version 21.05.1 (usegalaxy.org). Individual sequencing files for each condition within a replicate were concatenated and processed using the fastp application to filter low quality reads, trim reads and cut adapter sequences. Sequences were then aligned to either the C. trachomatis 434/Bu (ASM6858v1) genome assembly or the Homo sapiens GRCh38 genome assembly using HISAT2 (60). Read counts were generated using htseq-count (61) and output files were exported and compiled for differential gene expression analysis by DESeq2 in R (62). Principal-component analysis was performed on the regularized log-transformed count data. Volcano plots were generated using the EnhancedVolcano R package (63). Gene set enrichment analysis for KEGG pathways was conducted using the clusterProfiler R package (64). Mapping of gene expression data to KEGG pathways was performed using the Pathview package in R (65). Gene network maps were generated by submitting gene lists to the STRING database (66) and then formatting networks in Cytoscape (67). Note that any chlamydial genes not recognized by STRING were automatically filtered out during analysis.

### Quantitative PCR.

All quantitative PCR (qPCR) assays were performed using Power Up SYBR Green Master Mix (Applied Biosystems, Thermo Fisher Scientific) essentially as previously described (23, 24). In brief, cDNA was diluted 1:5-1:10 and gDNA was diluted 1:50-1:100 in nuclease-free H_2_O (dilutions were identical within each experiment). The 2X PCR master mix was diluted to 1X in nuclease-free H_2_O with specific primers diluted to 500 nM (see Data set S4 for complete list of primers). To 79 μL of the master mix solution, 3.3 μL of template (cDNA or gDNA) was added and then aliquoted into three 25 μL technical replicate reactions in a 96-well optical plate. Reactions were analyzed on a QuantStudio 3 real-time PCR system with standard SYBR cycling conditions. All assays were performed with a melt-curve analysis to ensure specific product amplification across samples. Primer sets (Supplementary File 1) used in qPCR were validated against a standard curve of C. trachomatis L2 gDNA diluted from 2 × 10^−3^ to 2 × 10° ng per reaction. C_t_ values generated from each experimental reaction were then fit to a standard curve and only primer sets with an efficiency of 100% +/−5% were used.

Genome equivalents (GE) were calculated by first converting the mean C_t_ of the triplicate technical replicate reactions to a ng quantity of gDNA (ng template) with the linear equation generated from the standard curve of the *euo* primer pair. This value was then normalized to the total ng/μL gDNA isolated for each sample as follows:

$$\mathrm{GE}=\frac{\mathrm{ng}\;\mathrm{template}}{\frac{\mathrm{ng}}{\text{μ}L}\;\mathrm{gDNA}}$$

For the quantification of transcript expression by RT-qPCR, a transcriptome-based normalization was used based on the geometric average of multiple control genes, which were empirically determined using the geNorm method (68). For more information on the geNorm analysis, see Text S1. In brief, all transcript expression data was normalized to the geometric mean of the expression of *groEL_1*, *euo*, *nrdA*, and *nrdB*. The ΔΔCt method was then used to determine relative expression values and the log_2_-transformed fold change was analyzed to facilitate comparisons between conditions where the magnitude of gene expression changed considerably (e.g., expression of the *trpRBA* operon in iron- or tryptophan-starved conditions). Thus, transcript expression (TE) was calculated as:

$$\mathrm{TE}=2^{-(\left[Ct_{\mathrm{Exp}}-Ct_{\mathrm{geNorm}}]-Ct_{\mathrm{Ref}}\right)}$$

Where C_t_(Exp) is the C_t_ value of the experimental gene being analyzed, C_t_(geNorm) is the geometric mean of the C_t_ values for the control genes, and C_t_(Ref) is the mean C_t_ value of the reference condition, in this case, UTD24. All C_t_ values were corrected for dilution prior to the computation of transcript expression.

### Immunofluorescent confocal microscopy.

To analyze inclusion morphology, HeLa cells were seeded onto acid-washed glass coverslips in 24-well tissue culture plates and infected at MOI = 5. At the indicated times postinfection, coverslips were washed with phosphate-buffered saline (PBS) and cells were fixed with 4% paraformaldehyde in PBS for 15 min at room temperature. Fixation solution was then aspirated and coverslips were either stored at 4°C in PBS or immediately processed for immunofluorescence assays by permeabilizing cells in PBS + 0.2% Triton X-100 (Thermo Scientific) for 10 min at room temperature with rocking. Permeabilization solution was then decanted, and coverslips were washed 3x with PBS. Coverslips were blocked in PBS + 3% bovine serum albumin (BSA) for 1 h at room temperature with rocking. Coverslips were then washed 3x with PBS prior to being overturned on a 50 μL droplet of PBS + 3% BSA containing primary antibody diluted 1:1000. To detect chlamydial GroEL, cells were stained with monoclonal mouse anti-cHsp60 (MA3-023, Invitrogen, ThermoFisher Scientific). Coverslips were incubated on primary antibody solution overnight at 4°C in an opaque humidified container. Coverslips were then washed thoroughly by repeated submersion (~50x) in 100 mL PBS before being overturned on a 50 μL droplet of PBS + 3% BSA + 1:1000 secondary antibody + 2.5 μg/mL 4′,6-diamidino-2-phenylindole (DAPI) to label nuclei. A donkey anti-mouse Alexa Fluor-594 secondary antibody (Invitrogen, Thermo Fisher Scientific) was used to label the primary mouse anti-cHsp60. Coverslips were then incubated for at least 1 h at room temperature in an opaque humidified container prior to being washed as described above in Milli-Q H_2_O, and then being mounted on glass microscope slides with 10 μL Shandon Immu-Mount (Thermo Fisher Scientific). Mounting medium was allowed to solidify overnight. Confocal microscopy was performed on a Nikon Ti2 Eclipse spinning-disk confocal microscope. All images were acquired using identical laser power and exposure settings. To enhance visualization of inclusion morphology, contrast and brightness were adjusted as necessary for each condition in Fiji ImageJ (69). All images are summed Z-projections of Z-stacks spanning the entire depth of the inclusions in the field.

### Reinfection assay.

At the indicated times postinfection for the relevant treatment conditions, infected cells were scraped into cell culture media and collected in 2 mL microcentrifuge tubes. Cell suspensions were then centrifuged at 21,000 × *g* for 30 min at 4°C to rupture the cells. The supernatant was aspirated, and the cell pellet was resuspended in 500 μL of sterile-filtered Sucrose-phosphate-glutamate (SPG; 220 mM sucrose, 10 mM Na_2_HPO_4_, 4 mM KH_2_PO_4_, 5 mM Glutamic Acid) buffer. The resuspended cell lysate was centrifuged at 200 × *g* for 5 min at 4°C to pellet cell debris. The supernatant was stored at −80°C and used to reinfect a confluent HeLa cell monolayer in one well of a 24-well tissue culture plate in a 10-fold dilution series starting at 10 or 100 μL of inoculum. At 24 hpi, the reinfected cells were fixed and stained as above for DAPI and GroEL, and at the appropriate dilution for each condition, inclusions were enumerated per field (total of 5 fields per replicate), and the number of IFU per mL of inoculum was calculated. For reactivation, media containing mizoribine was removed at 24 hpi and the samples were incubated with fresh media for an additional 16 h prior to sample collection. The limit of detection was calculated to be one inclusion identified per field at 100 μL of inoculum.

### Measurement of intracellular GTP levels.

Infected or mock-infected cells under the indicated treatment conditions were collected at 24 hpi by washing cells in 2 mL PBS, aspirating the wash buffer, and then scraping the cells into 250 μL 1% trichloracetic acid (TCA) solution to precipitate macromolecular complexes. The lysate was centrifuged to collect precipitates and the supernatant was neutralized to pH ~7.5 with 20 μL 1 M Tris-HCl, pH 8.5 prior to storage at -80°C. Intracellular GTP levels were then measured using the GTPase-Glo assay kit (Promega Corporation), which was adapted to measure GTP from cell lysates. In brief, 60 μL of TCA-precipitated lysate was diluted in 60 μL of GTPase/GAP buffer. A 10 μM stock solution of rGTP, provided by the manufacturer, was used as a positive control for the assay. Each sample was then aliquoted in quadruplicate 25 μL volumes in separate wells of a white polystyrene 96-well plate. Two wells for each sample received GTPase-Glo buffer containing ADP and GTPase-Glo reagent ([ATP]+[GTP]), while the other 2 wells received GTPase-Glo buffer alone ([ATP]). Samples were then incubated for 30 min at room temperature with shaking. Following incubation, 50 μL of Detection reagent was added to each well and allowed to incubate for another 10 min at room temperature with shaking. Luminescence was then measured on a Tecan Spark microplate reader (Tecan Group Ltd.). GTP levels were calculated by subtracting the baseline ([ATP]) luminescence reading from the converted ([GTP]+[ATP]) luminescence reading. All values were normalized to the mean of the untreated, mock-infected control group.

### Immunofluorescent analysis of chlamydial inclusion size.

Confluent HeLa cell monolayers were infected at MOI = 1 for 8 h prior to treatment with 50 μM mizoribine or 2,2-bipyridyl either alone or in combination in parallel with a mock-treated control. Treatment was allowed to proceed until 24 hpi (16 h). Fixation and staining with DAPI and anti-cHsp60 was performed as described above. Where possible, five single z-plane fields were acquired per condition on a Nikon Ti2 Eclipse spinning disk confocal microscope with a 60x objective. Inclusion size was determined in Fiji ImageJ. To guarantee even sample sizes for each condition, 15 values were randomly selected from each biological replicate and analyzed statistically as described in the relevant figure legend.

### Statistics.

All statistical computations were performed in RStudio (version 1.3.1093) using base platform functions and the code is available as indicated above. All plots were made in the ggplot2 base package (version 3.1.0) (70) and the ggpubr package (version 0.2.3; https://CRAN.R-project.org/package=ggpubr) or in Adobe Illustrator (version 24.1.2). All tests are indicated in the figure legends along with the value of N (independent biological replicates or observations). All plots represent the mean and standard deviation of the data, or the median and absolute median deviation. Significance was defined as a *P*-value below 0.05 and a sample size of 3 was considered satisfactory for estimating normality.

### Data availability.

All sequencing data generated in this study have been deposited at the NCBI Gene Expression Omnibus (GEO; Accession number: GSE179003) and are publicly available as of the date of publication. All other source data and original code for the analysis of RNA-sequencing data sets and other experimental data have been deposited at Mendeley Data (doi:10.17632/y872vkmbmj.2), and are publicly available as of the date of publication. Microscopy data reported in this paper will be shared by the corresponding author upon request. Any additional information required to reanalyze the data reported in this paper is available from the corresponding author upon request.

10.1128/mbio.02719-22.10 TEXT S1 Details of RT-qPCR normalization by the geNorm method. Download Text S1, DOCX file, 0.02 MB. Copyright © 2022 Pokorzynski et al. 2022 Pokorzynski et al. https://creativecommons.org/licenses/by/4.0/ This content is distributed under the terms of the Creative Commons Attribution 4.0 International license.

## References

[1] Prentice AM, Ghattas H, Cox SE. 2007. Host-pathogen interactions: can micronutrients tip the balance? J Nutr 137:1334–1337. doi:10.1093/jn/137.5.1334. 17449601

[2] Shtrichman R, Samuel CE. 2001. The role of gamma interferon in antimicrobial immunity. Curr Opin Microbiol 4:251–259. doi:10.1016/s1369-5274(00)00199-5. 11378475

[3] Finlay BB, McFadden G. 2006. Anti-immunology: evasion of the host immune system by bacterial and viral pathogens. Cell 124:767–782. doi:10.1016/j.cell.2006.01.034. 16497587

[4] Hood MI, Skaar EP. 2012. Nutritional immunity: transition metals at the pathogen–host interface. Nat Rev Microbiol 10:525–537. doi:10.1038/nrmicro2836. 22796883 PMC3875331

[5] Abreu R, Essler L, Giri P, Quinn F. 2020. Interferon-gamma promotes iron export in human macrophages to limit intracellular bacterial replication. PLoS One 15:e0240949. doi:10.1371/journal.pone.0240949. 33290416 PMC7723272

[6] Nairz M, Haschka D, Demetz E, Weiss G. 2014. Iron at the interface of immunity and infection. Front Pharmacol 5:1–10. doi:10.3389/fphar.2014.00152. 25076907 PMC4100575

[7] Byrne GI, Lehmann LK, Landry GJ. 1986. Induction of tryptophan catabolism is the mechanism for gamma-interferon-mediated inhibition of intracellular *Chlamydia psittaci* replication in T24 cells. Infect Immun 53:347–351. doi:10.1128/iai.53.2.347-351.1986. 3089936 PMC260881

[8] AbdelRahman YM, Belland RJ. 2005. The chlamydial developmental cycle. FEMS Microbiol Rev 29:949–959. doi:10.1016/j.femsre.2005.03.002. 16043254

[9] Wyrick PB. 2010. *Chlamydia trachomatis* persistence *in vitro*: an overview. J Infect Dis 201:88–95. doi:10.1086/652394. PMC2878585 20470046

[10] Panzetta ME, Valdivia RH, Saka HA. 2018. Chlamydia persistence: a survival strategy to evade antimicrobial effects *in-vitro* and *in-vivo*. Front Microbiol 9:1–11. doi:10.3389/fmicb.2018.03101. 30619180 PMC6299033

[11] Skilton RJ, Cutcliffen LT, Barlow D, Wang Y, Salim O, Lambden PR, Clarke IN. 2009. Penicillin induced persistence in *Chlamydia trachomatis*: high quality time lapse video analysis of the developmental cycle. PLoS One 4:e7723. doi:10.1371/journal.pone.0007723. 19893744 PMC2769264

[12] Belland RJ, Nelson DE, Virok D, Crane DD, Hogan D, Sturdevant D, Beatty WL, Caldwell HD. 2003. Transcriptome analysis of chlamydial growth during IFN-gamma-mediated persistence and reactivation. Proc Natl Acad Sci USA 100:15971–15976. doi:10.1073/pnas.2535394100. 14673075 PMC307677

[13] Hatch ND, Ouellette SP. 2020. Inhibition of tRNA synthetases induces persistence in Chlamydia. Infect Immun (March) 88:e00943-19. doi:10.1128/IAI.00943-19. PMC7093126 31964747

[14] Beatty WL, Belanger TA, Desai AA, Morrison RP, Byrne GI. 1994. Tryptophan depletion as a mechanism of gamma interferon-mediated chlamydial persistence. Infect Immun 62:3705–3711. doi:10.1128/iai.62.9.3705-3711.1994. 8063385 PMC303021

[15] Raulston JE. 1997. Response of Chlamydia trachomatis serovar E to iron restriction *in vitro* and evidence for iron-regulated chlamydial proteins. Infect Immun 65:4539–4547. doi:10.1128/iai.65.11.4539-4547.1997. 9353031 PMC175652

[16] Thompson CC, Carabeo RA. 2011. An optimal method of iron starvation of the obligate intracellular pathogen, *Chlamydia trachomatis*. Front Microbiol 2:1–10. doi:10.3389/fmicb.2011.00020. 21687412 PMC3109288

[17] Ardissone S, Scherler A, Pillonel T, Martin V, Kebbi-Beghdadi C, Greub G. 2020. Transcriptional landscape of *Waddlia chondrophila* aberrant bodies induced by iron starvation. Microorg 8:1848. doi:10.3390/microorganisms8121848. PMC7760296 33255276

[18] Beatty WL, Morrison RP, Byrne GI. 1995. Reactivation of persistent *Chlamydia trachomatis* infection in cell culture. Infect Immun 63:199–205. doi:10.1128/iai.63.1.199-205.1995. 7806358 PMC172978

[19] Wyrick PB, Knight ST. 2004. Pre-exposure of infected human endometrial epithelial cells to penicillin in vitro renders *Chlamydia trachomatis* refractory to azithromycin. J Antimicrob Chemother 54:79–85. doi:10.1093/jac/dkh283. 15163653

[20] Phillips-Campbell R, Kintner J, Schoborg RV. 2014. Induction of the *Chlamydia muridarum* stress/persistence response increases azithromycin treatment failure in a murine model of infection. Antimicrob Agents Chemother 58:1782–1784. doi:10.1128/AAC.02097-13. 24342653 PMC3957849

[21] Al-Younes HM, Rudel T, Brinkmann V, Szczepek AJ, Meyer TF. 2001. Low iron availability modulates the course of *Chlamydia pneumoniae* infection. Cell Microbiol 3:427–437. doi:10.1046/j.1462-5822.2001.00125.x. 11422085

[22] Ouellette SP, Rueden KJ, Rucks EA. 2016. Tryptophan codon-dependent transcription in chlamydia pneumoniae during gamma interferon-mediated tryptophan limitation. Infect Immun 84:2703–2713. doi:10.1128/IAI.00377-16. 27400720 PMC4995898

[23] Pokorzynski ND, Brinkworth AJ, Carabeo R. 2019. A bipartite iron-dependent transcriptional regulation of the tryptophan salvage pathway in *Chlamydia trachomatis*. Elife 8:e42295. doi:10.7554/eLife.42295. 30938288 PMC6504234

[24] Pokorzynski ND, Hatch ND, Ouellette SP, Carabeo RA. 2020. The iron-dependent repressor YtgR is a tryptophan-dependent attenuator of the trpRBA operon in *Chlamydia trachomatis*. Nat Commun 11:1–15. doi:10.1038/s41467-020-20181-5. 33353937 PMC7755916

[25] Hayward RJ, Humphrys MS, Huston WM, Myers GSA. 2021. Dual RNA-seq analysis of *in vitro* infection multiplicity and RNA depletion methods in *Chlamydia*-infected epithelial cells. Sci Rep 11:1–14. doi:10.1038/s41598-021-89921-x. 34001998 PMC8128910

[26] Eickhoff M, Thalmann J, Hess S, Martin M, Laue T, Kruppa J, Brandes G, Klos A. 2007. Host cell responses to *Chlamydia pneumoniae* in Gamma interferon-induced persistence overlap those of productive infection and are linked to genes involved in apoptosis, cell cycle, and metabolism. Infect Immun 75:2853–2863. doi:10.1128/IAI.01045-06. 17353287 PMC1932845

[27] Thompson CC, Nicod SS, Malcolm DS, Grieshaber SS, Carabeo RA. 2012. Cleavage of a putative metal permease in *Chlamydia trachomatis* yields an iron-dependent transcriptional repressor. Proc Natl Acad Sci USA 109:10546–10551. doi:10.1073/pnas.1201398109. 22689982 PMC3387080

[28] Carrasco JA, Tan C, Rank RG, Hsia R, Bavoil PM. 2011. Altered developmental expression of polymorphic membrane proteins in penicillin-stressed *Chlamydia trachomatis*. Cell Microbiol 13:1014–1025. doi:10.1111/j.1462-5822.2011.01598.x. 21504531 PMC3116966

[29] Becker E, Hegemann JH. 2014. All subtypes of the Pmp adhesin family are implicated in chlamydial virulence and show species-specific function. Microbiologyopen 3:544–556. doi:10.1002/mbo3.186. 24985494 PMC4287181

[30] Barry CE, III, Brickman TJ, Hackstadt T. 1993. Hc1-mediated effects on DNA structure: a potential regulator of chlamydial development. Mol Microbiol 9:273–283. doi:10.1111/j.1365-2958.1993.tb01689.x. 8412680

[31] Hackstadt T, Baehr W, Ying Y. 1991. *Chlamydia trachomatis* developmentally regulated protein is homologous to eukaryotic histone H1. Proc Natl Acad Sci USA 88:3937–3941. doi:10.1073/pnas.88.9.3937. 2023942 PMC51568

[32] Ouellette SP, Hatch TP, AbdelRahman YM, Rose LA, Belland RJ, Byrne GI. 2006. Global transcriptional upregulation in the absence of increased translation in *Chlamydia* during IFNγ-mediated host cell tryptophan starvation. Mol Microbiol 62:1387–1401. doi:10.1111/j.1365-2958.2006.05465.x. 17059564

[33] Scidmore MA, Hackstadt T. 2001. Mammalian 14-3-3β associates with the *Chlamydia trachomatis* inclusion membrane via its interaction with IncG. Mol Microbiol 39:1638–1650. doi:10.1046/j.1365-2958.2001.02355.x. 11260479

[34] Olson-Wood MG, Jorgenson LM, Ouellette SP, Rucks EA. 2021. Inclusion membrane growth and composition are altered by overexpression of specific inclusion membrane proteins in *Chlamydia trachomatis* L2. Infect Immun 89:e00094-21. doi:10.1128/IAI.00094-21. 33875478 PMC8208519

[35] Grieshaber NA, Fischer ER, Mead DJ, Dooley CA, Hackstadt T. 2004. Chlamydial histone-DNA interactions are disrupted by a metabolite in the methylerythritol phosphate pathway of isoprenoid biosynthesis. Proc Natl Acad Sci USA 101:7451–7456. doi:10.1073/pnas.0400754101. 15123794 PMC409939

[36] Brockett MR, Liechti GW. 2021. Persistence alters the interaction between *Chlamydia trachomatis* and its host cell. Infect Immun 89:e00685-20. doi:10.1128/IAI.00685-20. 34001559 PMC8281235

[37] Wood H, Fehlner-Gardner C, Berry J, Fischer E, Graham B, Hackstadt T, Roshick C, McClarty G. 2003. Regulation of tryptophan synthase gene expression in *Chlamydia trachomatis*. Mol Microbiol 49:1347–1359. doi:10.1046/j.1365-2958.2003.03638.x. 12940992

[38] Tipples G, McClarty G. 1993. The obligate intracellular bacterium *Chlamydia trachomatis* is auxotrophic for three of the four ribonucleoside triphosphates. Mol Microbiol 8:1105–1114. doi:10.1111/j.1365-2958.1993.tb01655.x. 8361355

[39] McClarty G, Fan H. 1993. Purine metabolism by intracellular *Chlamydia psittaci*. J Bacteriol 175:4662–4669. doi:10.1128/jb.175.15.4662-4669.1993. 8335625 PMC204917

[40] Adams NE, Thiaville JJ, Proestos J, Juárez-Vázquez AL, McCoy AJ, Barona-Gómez F, Iwata-Reuyl D, de Crécy-Lagard V, Maurelli AT. 2014. Promiscuous and adaptable enzymes fill “Holes” in the tetrahydrofolate pathway in *Chlamydia* species. mBio 5:e01378-14. doi:10.1128/mBio.01378-14. 25006229 PMC4161248

[41] de Crécy-Lagard V, El Yacoubi B, de la Garza RD, Noiriel A, Hanson AD. 2007. Comparative genomics of bacterial and plant folate synthesis and salvage: predictions and validations. BMC Genomics 8:1–15. doi:10.1186/1471-2164-8-245. 17645794 PMC1971073

[42] Yokota S. 2002. Mizoribine: mode of action and effects in clinical use. Pediatr Int 44:196–198. doi:10.1046/j.1328-8067.2002.01536.x. 11896886

[43] Turka LA, Dayton J, Sinclair G, Thompson CB, Mitchell BS. 1991. Guanine ribonucleotide depletion inhibits T cell activation. Mechanism of action of the immunosuppressive drug mizoribine. J Clin Invest 87:940–948. doi:10.1172/JCI115101. 1999502 PMC329885

[44] Hoxhaj G, Hughes-Hallett J, Timson RC, Ilagan E, Yuan M, Asara JM, Ben-Sahra I, Manning BD. 2017. The mTORC1 signaling network senses changes in cellular purine nucleotide levels. Cell Rep 21:1331–1346. doi:10.1016/j.celrep.2017.10.029. 29091770 PMC5689476

[45] Calise SJ, Abboud G, Kasahara H, Morel L, Chan EKL. 2018. Immune response-dependent assembly of IMP dehydrogenase filaments. Front Immunol 9:1–15. doi:10.3389/fimmu.2018.02789. 30555474 PMC6283036

[46] Anthony SA, Burrell AL, Johnson MC, Duong-Ly KC, Kuo Y-M, Simonet JC, Michener P, Andrews A, Kollman JM, Peterson JR. 2017. Reconstituted IMPDH polymers accommodate both catalytically active and inactive conformations. Mol Biol Cell 28:2600–2608. doi:10.1091/mbc.e17-04-0263. 28794265 PMC5620369

[47] Itakura M, Holmes EW. 1979. Human amidophosphoribosyltransferase. An oxygen-sensitive iron-sulfur protein. J Biol Chem 254:333–338. doi:10.1016/S0021-9258(17)37922-X. 762062

[48] Shakoury-Elizeh M, Tiedeman J, Rashford J, Ferea T, Demeter J, Garcia E, Rolfes R, Brown PO, Botstein D, Philpott CC. 2004. Transcriptional remodeling in response to iron deprivation in *Saccharomyces cerevisiae*. Mol Biol Cell 15:1233–1243. doi:10.1091/mbc.e03-09-0642. 14668481 PMC363115

[49] Philpott CC, Protchenko O. 2008. Response to iron deprivation in *Saccharomyces cerevisiae*. Eukaryot Cell 7:20–27. doi:10.1128/EC.00354-07. 17993568 PMC2224162

[50] Finethy R, Coers J. 2016. Sensing the enemy, containing the threat: cell-autonomous immunity to *Chlamydia trachomatis*. FEMS Microbiol Rev 40:875–893. doi:10.1093/femsre/fuw027. 28201690 PMC5975928

[51] Taylor MW, Feng GS. 1991. Relationship between interferon-gamma, indoleamine 2,3-dioxygenase, and tryptophan catabolism. FASEB J 5:2516–2522. doi:10.1096/fasebj.5.11.1907934. 1907934

[52] Ibana JA, Belland RJ, Zea AH, Schust DJ, Nagamatsu T, AbdelRahman YM, Tate DJ, Beatty WL, Aiyar AA, Quayle AJ. 2011. Inhibition of indoleamine 2,3-dioxygenase activity by levo-1-methyl tryptophan blocks gamma interferon-induced chlamydia trachomatis persistence in human epithelial cells. Infect Immun 79:4425–4437. doi:10.1128/IAI.05659-11. 21911470 PMC3257928

[53] Ivashkiv LB. 2018. IFNγ: signalling, epigenetics and roles in immunity, metabolism, disease and cancer immunotherapy. Nat Rev Immunol 18:545–558. doi:10.1038/s41577-018-0029-z. 29921905 PMC6340644

[54] Su X, Yu Y, Zhong Y, Giannopoulou EG, Hu X, Liu H, Cross JR, Rätsch G, Rice CM, Ivashkiv LB. 2015. Interferon-γ regulates cellular metabolism and mRNA translation to potentiate macrophage activation. Nat Immunol 16:838–849. doi:10.1038/ni.3205. 26147685 PMC4509841

[55] Rother M, Gonzalez E, Teixeira da Costa AR, Wask L, Gravenstein I, Pardo M, Pietzke M, Gurumurthy RK, Angermann J, Laudeley R, Glage S, Meyer M, Chumduri C, Kempa S, Dinkel K, Unger A, Klebl B, Klos A, Meyer TF. 2018. Combined human genome-wide RNAi and metabolite analyses identify IMPDH as a host-directed target against *Chlamydia* infection. Cell Host Microbe 23:661–671. doi:10.1016/j.chom.2018.04.002. 29706504

[56] Paavonen J, Turzanski Fortner R, Lehtinen M, Idahl A. 2021. *Chlamydia trachomatis*, pelvic inflammatory disease, and epithelial ovarian cancer. J Infect Dis 224:S121–S127. doi:10.1093/infdis/jiab017. 34396414

[57] Dean D, Suchland RJ, Stamm WE. 2000. Evidence for long-term cervical persistence of *Chlamydia trachomatis* by omp1 genotyping. J Infect Dis 182:909–916. doi:10.1086/315778. 10950788

[58] Triboulet S, Subtil A. 2019. Make it a sweet home: responses of *Chlamydia trachomatis* to the challenges of an intravacuolar lifestyle. Microbiol Spectr 7:1–11. doi:10.1128/microbiolspec.BAI-0005-2019. PMC11588157 30848236

[59] Caldwell HD, Kromhout J, Schachter J. 1981. Purification and partial characterization of the major outer membrane protein of *Chlamydia trachomatis*. Infect Immun 31:1161–1176. doi:10.1128/iai.31.3.1161-1176.1981. 7228399 PMC351439

[60] Kim D, Langmead B, Salzberg SL. 2015. HISAT: a fast spliced aligner with low memory requirements. Nat Methods 12:357–360. doi:10.1038/nmeth.3317. 25751142 PMC4655817

[61] Anders S, Pyl PT, Huber W. 2015. HTSeq—a Python framework to work with high-throughput sequencing data. Bioinformatics 31:166–169. doi:10.1093/bioinformatics/btu638. 25260700 PMC4287950

[62] Love MI, Huber W, Anders S. 2014. Moderated estimation of fold change and dispersion for RNA-seq data with DESeq2. Genome Biol 15:1–21. doi:10.1186/s13059-014-0550-8. PMC4302049 25516281

[63] Blighe K, Rana S, Lewis M. 2021. EnhancedVolcano: publication-ready volcano plots with enhanced colouring and labeling. doi:10.18129/B9.bioc.EnhancedVolcano.

[64] Yu G, Wang L-G, Han Y, He Q-Y. 2012. clusterProfiler: an R package for comparing biological themes among gene clusters. OMICS 16:284–287. doi:10.1089/omi.2011.0118. 22455463 PMC3339379

[65] Luo W, Brouwer C. 2013. Pathview: an R/Bioconductor package for pathway-based data integration and visualization. Bioinformatics 29:1830–1831. doi:10.1093/bioinformatics/btt285. 23740750 PMC3702256

[66] Szklarczyk D, Gable AL, Lyon D, Junge A, Wyder S, Huerta-Cepas J, Simonovic M, Doncheva NT, Morris JH, Bork P, Jensen LJ, Mering Cv. 2019. STRING v11: protein–protein association networks with increased coverage, supporting functional discovery in genome-wide experimental datasets. Nucleic Acids Res 47:D607–D613. doi:10.1093/nar/gky1131. 30476243 PMC6323986

[67] Shannon P, Markiel A, Ozier O, Baliga NS, Wang JT, Ramage D, Amin N, Schwikowski B, Ideker T. 2003. Cytoscape: a software environment for integrated models of biomolecular interaction networks. Genome Res 13:2498–2504. doi:10.1101/gr.1239303. 14597658 PMC403769

[68] Vandesompele J, De Preter K, Pattyn F, Poppe B, Van Roy N, De Paepe A, Speleman F. 2002. Accurate normalization of real-time quantitative RT-PCR data by geometric averaging of multiple internal control genes. Genome Biol 3:1–12. 10.1186/gb-2002-3-7-research0034 PMC126239 12184808

[69] Schindelin J, Arganda-Carreras I, Frise E, Kaynig V, Longair M, Pietzsch T, Preibisch S, Rueden C, Saalfeld S, Schmid B, Tinevez J-Y, White DJ, Hartenstein V, Eliceiri K, Tomancak P, Cardona A. 2012. Fiji: an open-source platform for biological-image analysis. Nat Methods 9:676–682. doi:10.1038/nmeth.2019. 22743772 PMC3855844

[70] Wickham H. 2016. ggplot2: Elegant graphics for data analysis. Springer-Verlag, New York. https://ggplot2.tidyverse.org.

