# Supplementary figures and images for: Host Cell Amplification of Nutritional Stress Contributes To Persistence in Chlamydia trachomatis
Source: mBio. 2022 Nov 15;13(6):e02719-22. doi: 10.1128/mbio.02719-22 (PMC9765610; doi:10.1128/mbio.02719-22)

| A | Step | Gene            | M (stability) |
|---|------|-----------------|---------------|
|   | 1    | <i>omcB</i>     | 0.17552546    |
|   | 2    | <i>16S rRNA</i> | 0.16175151    |
|   | 3    | <i>ompA</i>     | 0.10932164    |
|   | 4    | <i>nrdA</i>     | 0.0407112     |
|   | 5    | <i>nrdB</i>     | 0.03446746    |

**B** Gene stability measure

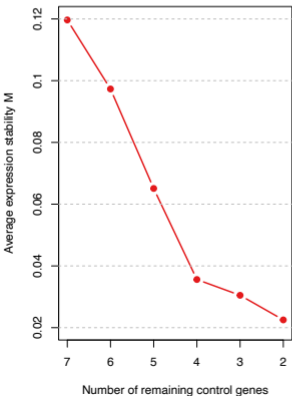

**C**

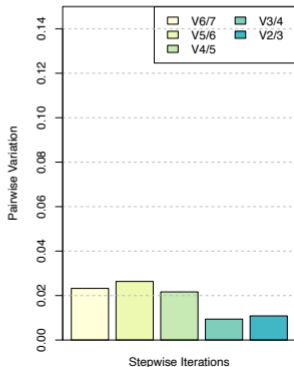

Supplement: FIG S1 [file mbio.02719-22-s0006.pdf]

*amiA*

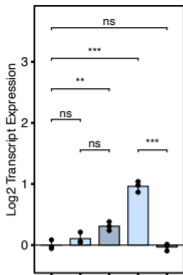

*incG*

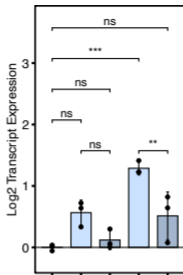

*incF*

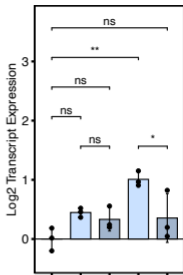

*sodM*

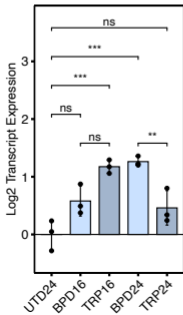

*ispE*

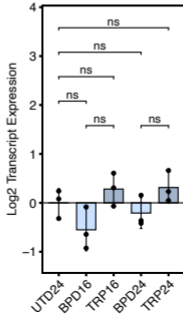

*tyrP*

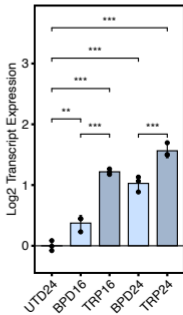

Supplement: FIG S2 [file mbio.02719-22-s0007.pdf]

Log2 Transcript Expression

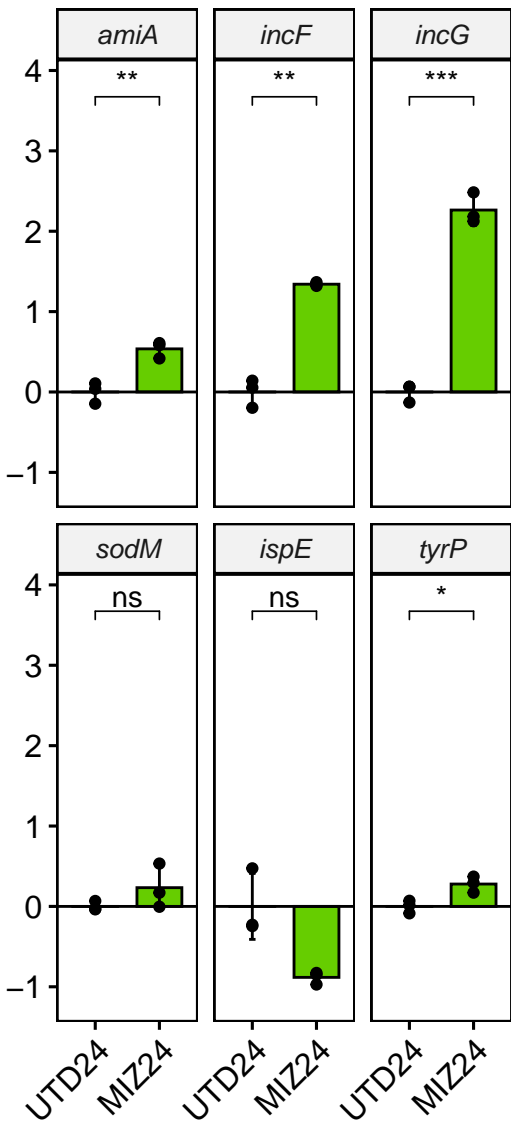

Supplement: FIG S4 [file mbio.02719-22-s0009.pdf]

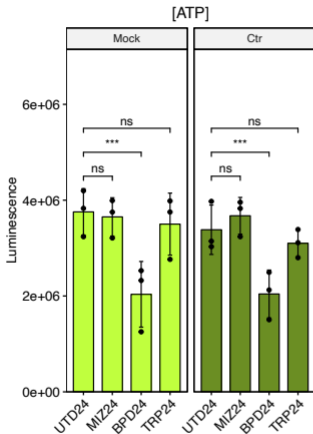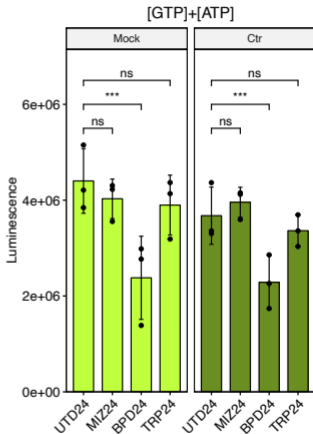

Supplement: FIG S5 [file mbio.02719-22-s0010.pdf]
