# Supplementary material for: Host Cell Amplification of Nutritional Stress Contributes To Persistence in Chlamydia trachomatis
Source: mBio. 2022 Nov 15;13(6):e02719-22. doi: 10.1128/mbio.02719-22 (PMC9765610; doi:10.1128/mbio.02719-22)
Supplement: FIG S3 [file mbio.02719-22-s0008.pdf]

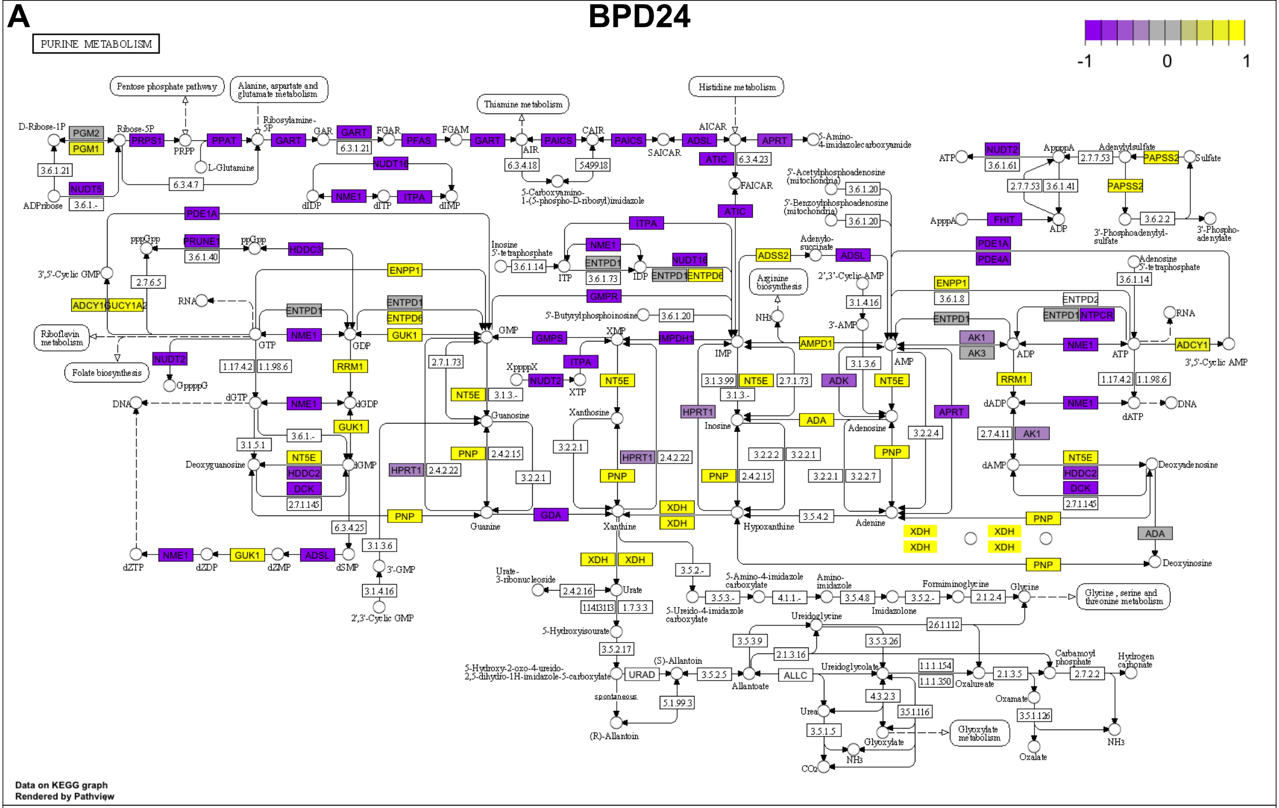
A
BPD24
-1
0
1
PURINE METABOLISM
Pentose phosphate pathway
Alanine, aspartate and glutamate metabolism
Thiamine metabolism
Histidine metabolism
Riboflavin metabolism
Folate biosynthesis
Arginine biosynthesis
Glycine, serine and threonine metabolism
Glyoxylate metabolism
Data on KEGG graph
Rendered by Pathview

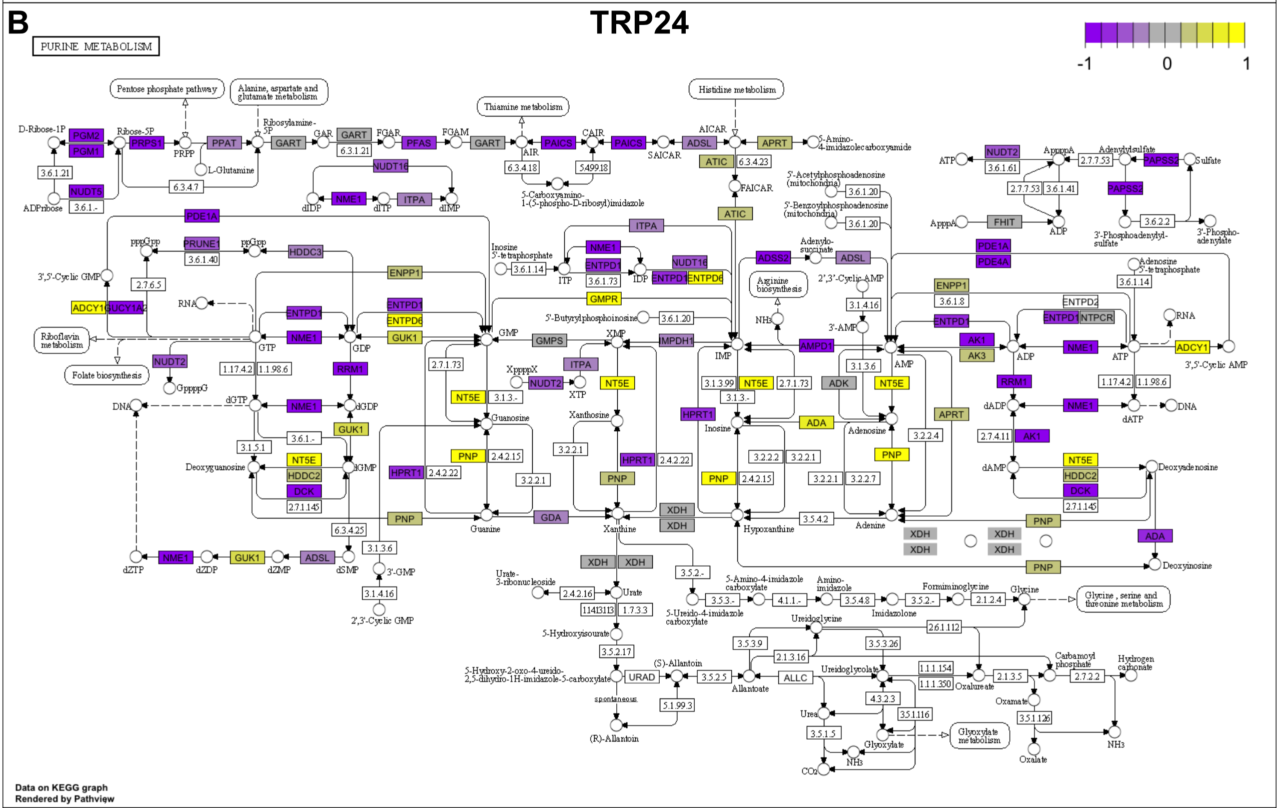
B
TRP24
-1
0
1
PURINE METABOLISM
Pentose phosphate pathway
Alanine, aspartate and glutamate metabolism
Thiamine metabolism
Histidine metabolism
Riboflavin metabolism
Folate biosynthesis
Arginine biosynthesis
Glycine, serine and threonine metabolism
Glyoxylate metabolism
Data on KEGG graph
Rendered by Pathview
