# Supplementary material for: Host Cell Amplification of Nutritional Stress Contributes To Persistence in Chlamydia trachomatis
Source: mBio. 2022 Nov 15;13(6):e02719-22. doi: 10.1128/mbio.02719-22 (PMC9765610; doi:10.1128/mbio.02719-22)
Supplement: TEXT S1 [file mbio.02719-22-s0005.docx]

**Supplemental Note 1**

The validation of RNA-sequencing data for chlamydial transcriptomes is not straight-forward (1, 2), primarily owing to the conventional normalization method utilized for targeted RT-qPCR gene expression data. Two general means of normalizing RT-qPCR data have been proposed: (1) normalization of gene expression to genome equivalents and (2) normalization of gene expression to the expression of an internal control gene. In the case of the former method, gene expression is interpreted on a per-organism basis, reflecting changes in absolute transcript levels. In the case of the latter method, gene expression is theoretically represented as a proportion of the total transcriptome, but the common practice of normalizing to a single control gene carries many assumptions that are often faulty, such as the turnover rate of the control gene under various conditions, leading to erroneous results (3). Yet, the nature of genome normalization can produce unclear results depending on the experimental question being asked. During chlamydial persistence, where genome copies are reduced and basal transcriptional activity increases (4), genome normalization can over-estimate the up-regulation of genes whose abundance as a proportion of the total transcriptome does not change.

We therefore considered an alternative means of RT-qPCR normalization: geometric averaging of multiple control genes by the geNorm method (5). This approach relies on the identification of stably expressed groups of control genes, empirically determined by an assessment of their stability (*i.e.* the maintenance of the ratio of their raw expression values) and their pairwise variation (*i.e.* the variation in stability between any two control genes across conditions). The analysis therefore provides a normalization factor that is based on the *stable relationship* of the expression of multiple control genes, rendering it more insensitive to instability or fluctuations in the expression of a single control gene. We used this method to analyze the following seven transcripts: *euo*, *omcB*, *groEL_1*, *ompA*, *nrdA*, *nrdB*, and 16S rRNA. We determined that across our experimental conditions, *euo* and *groEL_1* were the most stably associated genes under the tested conditions (Figure 1A-1B) and that the set of *euo, groEL_1*, *nrdA* and *nrdB* had the lowest average pairwise variation (Figure 1C) and were most suitable for the derivation of a normalization factor. We note that while the suggested cut-off for pairwise variation of a set of control genes is 0.15, we find that all genes analyzed here appear highly stable (with pairwise variation not exceeding 0.027), likely reflecting the strong effect of developmental regulation on the relationship of chlamydial gene expression. This normalization facilitated the confirmation of RNA-sequencing data by RT-qPCR by increasing the sensitivity for down-regulated or unchanged gene expression. This normalization also more accurately reflects the normalization methods utilized during RNA-sequencing, *i.e.* transcriptome-based normalization factors across conditions. We suggest that future gene expression studies in *Chlamydia* carefully consider the most suitable normalization method for the experimental question at hand.

**Supplemental References**

1. Ardissone S, et al. (2020) Transcriptional Landscape of Waddlia chondrophila Aberrant Bodies Induced by Iron Starvation. *Microorg*  8(12). doi:10.3390/microorganisms8121848.

2. Brinkworth AJ, Wildung MR, Carabeo RA (2018) Genomewide Transcriptional Responses of Iron-Starved Chlamydia trachomatis Reveal Prioritization of Metabolic Precursor Synthesis over Protein Translation. *mSystems* 3(1):e00184-17.

3. Engström P, Bailey L, Önskog T, Bergström S, Johansson J (2010) A comparative study of RNA and DNA as internal gene expression controls early in the developmental cycle of Chlamydia pneumoniae. *FEMS Immunol Med Microbiol* 58(2):244–253.

4. Ouellette SP, et al. (2006) Global transcriptional upregulation in the absence of increased translation in Chlamydia during IFNγ-mediated host cell tryptophan starvation. *Mol Microbiol* 62(5):1387–1401.

5. Vandesompele J, et al. (2002) Accurate normalization of real-time quantitative RT-PCR data by geometric averaging of multiple internal control genes. *Genome Biol* 3(7):research0034.1.
